# Meiofauna in the Gollum Channels and the Whittard Canyon, Celtic Margin—How Local Environmental Conditions Shape Nematode Structure and Function

**DOI:** 10.1371/journal.pone.0020094

**Published:** 2011-05-18

**Authors:** Jeroen Ingels, Alexei V. Tchesunov, Ann Vanreusel

**Affiliations:** 1 Marine Biology Department, Ghent University, Ghent, Belgium; 2 Department of Invertebrate Zoology, Faculty of Biology, Moscow State University, Vorobyovy Gory, Moscow, Russia; University of Aberdeen, United Kingdom

## Abstract

The Gollum Channels and Whittard Canyon (NE Atlantic) are two areas that receive high input of organic matter and phytodetritus from euphotic layers, but they are typified by different trophic and hydrodynamic conditions. Sediment biogeochemistry was analysed in conjunction with structure and diversity of the nematode community and differences were tested between study areas, water depths (700 m vs 1000 m), stations, and sediment layers. The Gollum Channels and Whittard Canyon harboured high meiofauna abundances (1054–1426 ind. 10 cm^−2^) and high nematode diversity (total of 181 genera). Next to enhanced meiofauna abundance and nematode biomass, there were signs of high levels of organic matter deposition leading to reduced sedimentary conditions, which in turn structured the nematode community. Striking in this respect was the presence of large numbers of ‘chemosynthetic’ *Astomonema* nematodes (*Astomonema southwardorum*, Order Monhysterida, Family Siphonolaimidae). This genus lacks a mouth, buccal cavity and pharynx and possesses a rudimentary gut containing internal, symbiotic prokaryotes which have been recognised as sulphur-oxidising bacteria. Dominance of *Astomonema* may indicate the presence of reduced environments in the study areas, which is partially confirmed by the local biogeochemical environment. The nematode communities were mostly affected by sediment layer differences and concomitant trophic conditions rather than other spatial gradients related to study area, water depth or station differences, pointing to small-scale heterogeneity as the main source of variation in nematode structure and function. Furthermore, the positive relation between nematode standing stocks, and quantity and quality of the organic matter was stronger when hydrodynamic disturbance was greater. Analogically, this study also suggests that structural diversity can be positively correlated with trophic conditions and that this relation is tighter when hydrodynamic disturbance is greater.

## Introduction

The Celtic Margin situated in the NE Atlantic is a highly productive system with significant primary production in the surface waters, which consequently supplies deep-sea sediments with high levels of organic matter (OM) and carbon [1], [2] compared to other deep-sea areas. This is especially the case for mid-slope depths, which are influenced by additional export from the shelf or upper slope, where sediment organic loads are higher [3]. At the Porcupine Seabight and further south along the Meriadzek Terrace, the margin is incised by numerous canyons and channels. These geohydrological structures provide conduits for the transport of sediment from the shelf to the abyssal plain and over-bank turbidity currents, which deposit on the intervening terraces and spurs [4], but they also accumulate high amounts of sediments and OM, making them important in budgeting carbon sinks and sources [5], [6]. In addition, at the Celtic Margin cascading of dense water masses down the slope is likely to occur [7] and may entrain fresh chlorophyll material rapidly down slope, as reported by Hill et al. [8]. These observations all imply the presence of enhanced levels of OM and phytodetrital material in canyons and channel systems in this area, which will have implications for the trophic conditions, the biogeochemical processes and ultimately the benthic fauna. In particular meiobenthic densities and standing stocks in canyons are under control of the flux of OM from surface productivity to the seafloor and resulting trophic conditions in the sediment [9], [10], [11]. In addition to enhanced (although variable) OM levels, canyons and channel systems generally display high hydrodynamic activity in the form of tidal currents, episodic slumps, turbidity flows, and density induced internal waves and cascading events, which are likely to have a significant effect on meiobenthos by physically disturbing the sediments they reside in [10], [11], [12], [13], [14], [15], [16], [17], [18].

The effect of natural disturbances on communities have continuously instigated ecologists to develop and test conceptual models that explain patterns at various temporal and spatial scales based on biological interactions and/or abiotic processes; e.g. Intermediate Disturbance Hypothesis [19], Dynamic Equilibrium Model [20]. In applying these models to the benthic environment, changes in sedimentary trophic parameters and physical disturbances may regulate deterministic biotic processes, leading to the large and small-scale patterns in benthic fauna. Specifically applied to the deep sea, Levin et al. [21] presented a schematic model based on five ecological forces (productivity, flow, bottom-water oxygen, sediment heterogeneity, biotic disturbance) to predict regional-scale variation of local diversity (1–10 m^2^). For the smaller benthic fauna, the meiofauna (32–1000 µm), one could even look at smaller spatial scales and include the vertical sediment profile in explaining community and diversity patterns. Such patterns along the vertical sediment profile may be explained by trophic conditions and oxygen concentration in the different sediment layers; e.g. TROX model for Foraminifera [22]. Following this concept, Soetaert et al. [23] suggested that similar processes may control benthic nematode community patterns. Based on various interpretations of environmental variation in the different schematic models it is likely that there is a hierarchical interaction between environmental drivers which regulates abundance and diversity of the meiofauna on various spatial and temporal scales.

Canyons and channel systems provide suitable habitats to test the effect of natural disturbances on benthic faunas. High organic loads and variable sedimentary trophic conditions, variable frequency and intensity of disturbance events, and sediment heterogeneity are all features ascribed to canyon and channel systems and may be used to explain benthic faunal patterns. Furthermore, it has become increasingly clear that variability between canyons and even within one canyon system is very high and that productivity and disturbance processes can shape meiobenthic communities differently [10], [11], [24]. Due to this high variability, studies investigating how processes that affect the sedimentary environment influence the benthic fauna in canyons and channel systems should be conducted on a scale that not only allows for sedimentary contrasts on a cm-scale, but also includes comparisons between different canyons or channels and water depths. The extent to which sedimentary conditions and processes control standing stocks, and structural and functional diversity of meiobenthic communities in canyons and channels would provide important information on how benthic communities are shaped and what drives diversity in these environments.

The Gollum Channel system and Whittard Canyon along the Irish Margin have only recently been subject to more intensive investigations, primarily within the framework of the EU FP6 HERMES project, and therefore little previous information is available [25], [26], especially on the biology of the benthos [27], [28]. In this first study on nematode communities – as representative for the metazoan meiofauna - from these two areas we tested nematode standing stocks, diversity, and community structure for differences between study sites, water depths, and sediment depths. Differences are interpreted in relation to biogeochemical parameters, trophic characteristics and sediment disturbance processes. Given the high habitat heterogeneity and environmental variability in these canyons and channels, we hypothesise that the nematode assemblages will be largely controlled by small-scale, local environmental conditions rather than large-scale differences between canyons or water depths.

## Materials and Methods

### Sampling and study area

**Figure 1:**
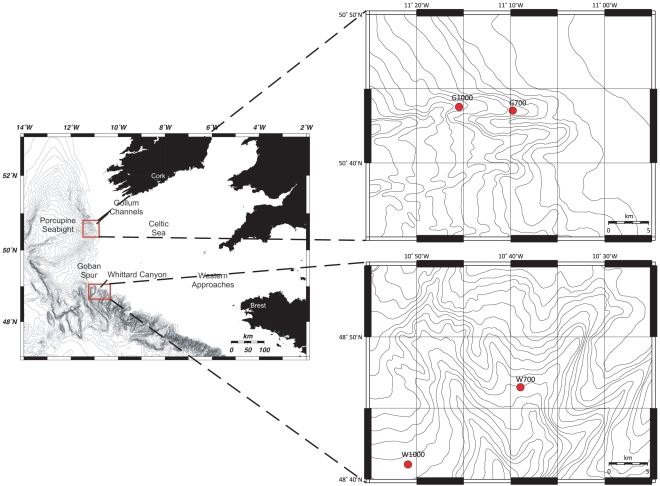
Overview (left) and detailed maps (right) of the study area with the Gollum stations (top right) and Whittard stations (bottom right). G1000: Gollum Channel at 1090 m depth, G700: Gollum Channel at 755 m depth, W1000: Whittard Canyon at 1160 m depth, W700: Whittard Canyon at 762 m depth.

During the HERMES RV *Belgica* 2006/13 cruise, 23–29 June 2006 [29] to the Irish Margin, meiofauna samples were taken using a Midicorer (MDC, Ocean Scientific International Ltd), yielding samples with a virtually undisturbed sediment-water interface. The MDC was equipped with four core tubes with an inner diameter of 100 mm, but 57 mm diameter subcores were taken for meiofauna and nematode analyses. Two sites in the Gollum Channel system were sampled at ca. 700 and 1000 m depth (G700 and G1000, respectively) as well as two stations in between two north-eastern branches of the Whittard Canyon at similar depths (W700 and W1000, respectively). An overview and details of the sampling areas are provided in Fig. 1.

The Gollum Channel system has been described as a tributary channel system incising the upper slope of the south-eastern Porcupine Seabight [30], [31]. It converges into one main channel, the Gollum Channel, which opens into the Porcupine Abyssal Plain. Samples were taken in the most northerly channel of the Baggins branch of the channel system, the Bilbo Channel [32]. The Gollum Channel system lies along the NE Atlantic Margin where the water mass characteristics in the upper 1000 m are dominated by Eastern North Atlantic Water and Mediterranean Outflow Water. This results in relatively warm (8–10°C) and saline water (ca. 35.5) between 700 and 1000 m depth, with weak vertical temperature and density gradients [33]. Hydrographic activity in the Bilbo Channel was investigated by White [33] and indicated mean currents of 4.7 cm s^−1^ with a maximum recorded speed of 53 cm s^−1^. The flow in the canyon channel is dominated by the semi-diurnal tide and is fairly dynamic, resulting in currents strong enough to produce significant turbidity. Like many other canyon and channel systems, there appears to be a significant down-slope flow of suspended material within the Bilbo Channel [34].

The Whittard Canyon consists of a number of deeply incised branches that extend from the shelf break south of the Goban Spur. Further down canyon, the walls are lower but the branching nature of this canyon continues towards the canyon fan at abyssal depths [26]. The sampling stations in the Whittard Canyon were situated on an interfluvial area covered in fine sediments in between two upper NE branches (Fig. 1). In the upper reaches of the canyon down-slope sediment transport is dominated by turbidity currents. These mud-rich flows are of sufficient size to overspill the canyon walls, leading to deposition of mainly fine sediments, e.g. muds and clays, on the adjacent continental slope [4]. Along the Celtic Shelf edge a mean northwestern along-slope flow of ca. 6 cm s^−1^ has been reported [35].

### Biogeochemistry

Each 1-cm-thick slice from three replicate cores at each sampling site (two for G700) were analysed for granulometric and geochemical parameters down to the 5-cm level. Grain size distribution was measured with a Malvern Mastersizer 2000 (0.02–2000 µm size range). For total sedimentary organic carbon (TOC) and nitrogen (TN), samples were lyophilised, homogenised and acidified with 1% HCl until complete decarbonisation and dried before being analysed with a PDZ Europa ANCA-GSL elemental analyser (UC Davis, USA). Chloroplastic Pigment Equivalents (CPE: sum of chlorophyll a (chl-a) and its degradation products (phaeopigments)) [36] served to estimate the amount of sedimentary OM derived from primary production. Sediment samples were lyophilised and homogenised, after which pigments were extracted in 90% acetone, separated using reverse-phase HPLC, and measured with a Gilson fluorescence detector according to Wright & Jeffrey [37]. The ratio chl-a∶phaeopigments was used as a measure of ‘freshness’ of the photosynthetically derived OM in the sediments, the ratio chl-a∶TOC gives an indication of the bioavailability of the bulk OM.

### Meiofauna and nematode analysis

At each station, three replicate samples were sliced per cm down to 5 cm sediment depth and fixed in buffered 4% formalin. Samples were washed over a 1000-µm mesh and then sieved on a 32-µm mesh to retain the meiofauna fraction. The meiofauna was then elutriated by the centrifugation-flotation technique using LUDOX [38], [39] and stained with Rose Bengal. All metazoan meiobenthic organisms were classified at higher taxon level following Higgins & Thiel [40] and counted under a stereo-microscope (50×magnification). For nematode analysis, 100–150 individuals were picked out randomly from each sediment slice and transferred using the formalin-ethanol-glycerine technique [38] and mounted on glass slides. Nematode identifications followed the pictorial key to nematode genera of Warwick et al. [41], taxonomic literature from the nematode library of Ghent University, and the NeMys database [42]. All individuals were grouped into four feeding types *sensu* Wieser [43]: selective deposit feeders (1A), non-selective deposit feeders (1B), epistratum feeders (2A), and predators/scavengers (2B). One additional feeding type was defined for gutless nematodes of the genus *Astomonema*. Because these nematodes have a degenerated alimentary canal with absence of mouth and buccal cavity, they do not belong to any of Wieser's four feeding types [43]. Length (excluding filiform tail tips) and maximal width were measured using a Leica DMR compound microscope and Leica LAS 3.3 imaging software. Nematode wet weight was calculated according to Andrassy's [44] formula and translated into nematode dry weight with a dry-to-wet ratio of 0.25 [39]. To calculate total nematode biomass associated with sediment layers, sampling stations and feeding types, average biomass was calculated for each nematode genus and multiplied by their density per sediment layer, for each layer, station and feeding type.

### Data analysis

The multivariate community data on genus level (standardised, square-root transformed, Bray-Curtis similarity was used to calculate resemblance) was analysed by means of non-parametric permutational ANOVA (PERMANOVA [45], [46]) to assess differences between the Gollum and Whittard areas, between water depths, and between sediment layers. The data set was analysed using a 4-factor mixed model design (factors: Canyon (Ca; fixed), Water Depth (WD; fixed), Sediment Depth (SD; fixed), Cores (Co; to account for replicate variability; random and nested in Ca×WD) in PERMANOVA+ for PRIMER [46]). Since the vertical layers in the sediment were not replicated within each core, a split-plot design was used leading to a repeated measures analysis whereby the main-factor test was followed by pair-wise comparisons within each Ca×WD combination to investigate significant interaction effects in the full-model test. A non-metrical Multi-dimensional scaling plot (MDS) was used to visualise the PERMANOVA results and illustrate the three-way interaction terms. The MDS was obtained by calculating the centroids of the three-way interaction cell groupings in the full multivariate Bray-Curtis space, followed by calculating the distances among them (PERMANOVA+ for PRIMER [46]). A two-way crossed (Ca,WD) univariate PERMANOVA test was performed on average biomass data (data was fourth-root transformed, resemblance calculated with Bray-Curtis). Averaging the biomass data over replicates gave a more realistic estimate of the true biomass per sediment layer, station and canyon.

A two-way crossed (factors: Canyon and Water Depth) SIMPER (Similarity Percentages – species contributions) analysis was performed to reveal which genera are responsible for the multivariate community patterns within and between canyons and water depths.

Structural diversity of the community was calculated using Hill's [47] diversity numbers (*H_0_*, *H_1_*, *H_2_*, *H_inf_*), estimated number of genera (EG(51), [48], [49]). Functional or trophic diversity (TD) was calculated as the reciprocal value of the trophic index (Θ^−1^) by Heip et al. [50], so that higher values correspond with higher TD, based on relative abundance data.

The full set of 18 available environmental variables was tested for collinearity (Draftsman plot and Spearman correlation matrix) and (redundant) variables with correlations (r^2^) >0.9 were omitted from the model; chl-a, chl-a∶phaeo, chl-a∶TOC,a nd CPE∶TOC needed log(X+0.1) transformation to compensate for skewness. For analysis of selected geochemical variables (chl-a, CPE, chl-a∶phaeo, chl-a∶TOC, TOC, TN, C∶N, mean grain size), separate univariate PERMANOVA tests were performed (data was normalised, Euclidean distance was used to calculate resemblance). The PERMANOVA design was identical to the one used for community analysis. RELATE and DISTML (distance-based linear model) routines were performed [51], [52] to analyse and model the relationship between the nematode genera assemblages and the environmental variables (chl-a, CPE, chl-a∶phaeo, chl-a∶TOC, CPE∶TOC, TOC, TN, C∶N, mean grain size). The assemblage DISTML was constructed using the step-wise selection procedure and the adjusted R^2^ as selection criterion to enable the fitting of the best explanatory environmental variables in the model [51]. In addition, DISTLM models were calculated for selected biotic paramaters (Nematode density, relative abundance ‘chemosynthetic’ *Astomonema* nematodes, total nematode biomass, biomass ‘chemosynthetic’ *Astomonema*, trophic diversity (TD), Hill's diversity numbers *H_0_*, *H_1_*, and expected number of genera (EG(51)). Euclidean distance was used as resemblance measure in all DISTLM procedures. Complementary to these analyses, non-parametric Kendall-Tau correlations were computed between the same selected biotic parameters and abiotic variables for each of the canyon/channel systems.

## Results

### Sediment characteristics

**Figure 2:**
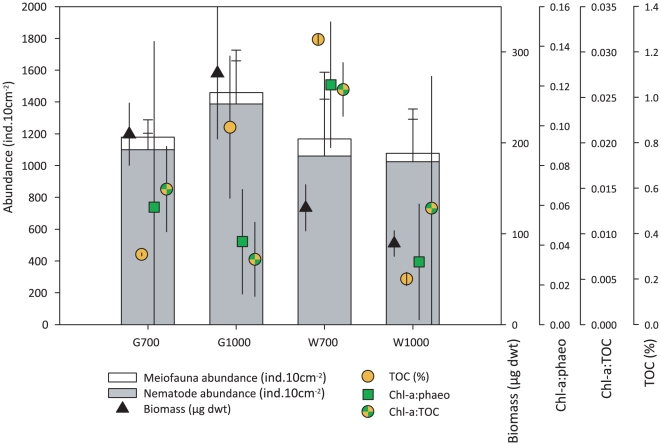
Average meiofauna and nematode abundance, nematode biomass, and selected sedimentary biogeochemical variables for each station (average total core values 0–5 cm, 10 cm^−2^). TOC: total sedimentary organic carbon, Chl-a∶phaeo: chlorophyll a divided by its degradation products (phaeophytines) indicating ‘freshness’ of the phytodetrital OM, chl-a∶TOC: chlorophyll a divided by total organic carbon content indicating bioavailability of the bulk OM, dwt: dry weight. G1000: Gollum Channel at 1090 m depth, G700: Gollum Channel at 755 m depth, W1000: Whittard Canyon at 1160 m depth, W700: Whittard Canyon at 762 m depth. Values averaged over replicates; error bars denote standard deviations.

**Figure 3:**
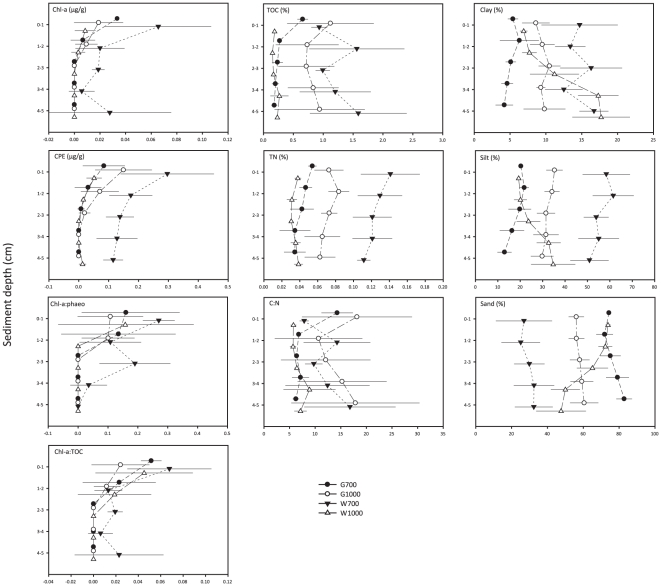
Average values for selected sedimentary biogeochemical parameters along the vertical sediment profile. Chl-a: chlorophyll a; CPE: chloroplastic pigment equivalents; Chl-a∶phaeo: chlorophyll a divided by its degradation products (phaeophytines) indicating ‘freshness’ of the phytodetrital OM; Chl-a∶TOC: chlorophyll a divided by total organic carbon content indicating bioavailability of the bulk OM; TOC: total organic carbon concentration; TN: total nitrogen concentration; C∶N: molar carbon/nitrogen ratio; Clay, Silt, Sand: volume percent clay, silt, sand content, respectively. G1000: Gollum Channel at 1090 m depth, G700: Gollum Channel at 755 m depth, W1000: Whittard Canyon at 1160 m depth, W700: Whittard Canyon at 762 m depth. Values averaged over replicates; error bars denote standard deviations.

Biogeochemical properties of the sediment are shown in Figs. 2 and 3. Grain-size fractions differed significantly between study areas and individual stations (p<0.01, Table S1). Highest muddy clay and silt content were observed at W700 and highest sand content at G700 (Fig. 3). At station W1000, the sediment became finer with increasing sediment depth, whilst at the other stations average grain size values declined slightly with sediment depth (Fig. 3).

Sedimentary TOC (p<0.05) and TN (p<0.01) contents differed significantly between stations (Ca×WD, Table S1). TN also differed significantly between canyons and water depths (p<0.05, Table S1), which was supported by significant pair-wise comparisons within each canyon or water depth group (data not shown). C∶N only differed significantly between stations (p<0.05, Table S1). Station W700 was characterised by highest average total-core TOC and TN content, followed by G1000, G700, and W1000, respectively (Fig. 2). At stations G1000 and W700, average total-core TOC, TN and C∶N values were higher compared to G700 and W1000 (Fig. 2), indicating higher amounts of OM with greater fractions of terrestrial and/or very degraded OM at these stations. No clear gradients were observed along the vertical sediment profile for TOC, TN, and C∶N (Fig. 3).

Chl-a differed significantly between canyon areas and water depths (p<0.05, Table S1), but the test showed a significant interaction term (Ca×WD), which indicates station differences (p<0.01, Table S1). Subsequent pair-wise comparisons within each canyon and water depth level showed that these were due to significant differences between W700 and the other stations (data not shown, but see Fig. 3). CPE levels contrasted significantly between water depths (p<0.05, Table S1), but these were also caused by contrasting values between W700 and the other stations. CPE and chl-a concentrations were clearly higher at W700 than at other stations (Fig. 2, 3) and decreased significantly with sediment depth at all stations (p<0.05, Table S1; Fig. 3). The ‘freshness’ (chl-a∶phaeo) and bio-availability (chl-a: TOC) of OM decreased significantly with sediment depth at each station (p<0.01, Table S1; Fig. 3), but did not contrast between canyon areas, water depths or stations (Table S1). Sediments at W700 showed higher chl-a∶phaeo and chl-a∶TOC compared to the other stations, indicating the presence of more ‘fresher’ and bio-available OM.

### Metazoan meiofauna

A total of 21 higher meiofauna groups were identified of which 20 occurred in the Gollum area and 15 in the Whittard area (Table S2). The most diverse station was G1000 with 18 different groups. Nematodes were consistently the dominant taxon (90.2–95.2%), followed by nauplius larvae (1.51–4.30%) and copepods (1.06–2.94%). Other taxa such as polychaetes and tardigrades occurred typically in lower abundances (<1%). Highest meiofauna abundance was recorded at G1000 (Table S2, Fig. 2), but was still similar to abundances at other stations because of high variability between replicates. Total meiofauna abundances consistently decreased with increasing sediment depth.

### Nematodes

#### Nematode community and biomass

**Figure 4:**
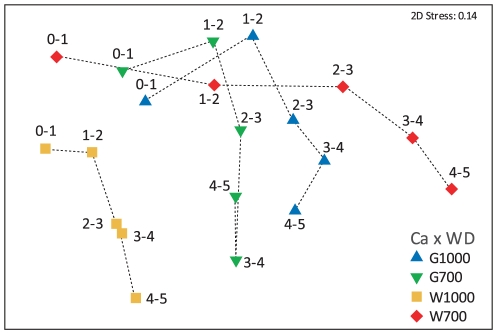
Non-metric MDS plot (Bray Curtis similarity) based on standardised and square-root transformed nematode genera relative abundance data. W700: Whittard 762 m water depth, W1000: Whittard 1160 m water depth, G700: Gollum 755 m water depth, G1000: Gollum 1090 m water depth. Labels denote sediment layer (cm). Dotted lines connect the succeeding sediment layers per station.

The PERMANOVA community results indicated significant differences between the Gollum and Whittard areas, water depths, sediment layers, and stations (p<0.01, Table 1). The significant three-way interaction term (Ca×WD×SD) called for pair-wise comparisons within each two-factor combination to investigate the nature of the effect (Table S3) and the MDS plot in Fig. 4 clearly shows the reasons for the three-way interaction suggested by the pair-wise comparisons. There is the pattern of a gradient across the different sediment layers from the upper left (0–1) to the lower right (4–5), but the size of the sediment-layer effect is very different in the different canyons and at different water depths (Fig. 4). More specifically, W1000 has a very modest sediment-layer effect, while W700 has a much larger effect, spreading practically all the way across the first MDS axis (Fig. 4). Overall, the surface layers (0–1 cm) are much more similar than the deeper layers, since all 0–1 cm points are more densely grouped. The difference between water depths is much greater for the Whittard samples than it is for the Gollum samples (Table S3, pair-wise comparisons within Ca×SD; Fig. 4) and at 1000 m water depth the differences between Gollum and Whittard are more prominent than at 700 m water depth (Table S3, pair-wise comparisons within WD×SD, Fig. 4). In general, the MDS plot illustrates clearly the extent to which differences between sediment layers exceed differences between canyons and water depths (Fig. 4), which is supported by the estimated components of variation (Table 1), and points to small-scale heterogeneity as the main source of variation in nematode structure. Moreover, the important role of sediment depth in shaping community composition was clearly illustrated by the clustering of 0–1 cm layers and the pronounced dissimilarity between the 4–5 cm layers from different stations (Fig. 4).

**Table 1:** Results from multivariate PERMANOVA analyses for differences in nematode community structure.

Source	df	SS	MS	Pseudo-F	P(perm)	Unique perms	Estim. of comp. of variation
Ca	1	10490	10490	48.268	***0.0021***	9826	285.89
WD	1	8657.3	8657.3	39.836	***0.0024***	9839	222.89
SD	4	23748	5936.9	5.474	***0.0001***	9879	415.91
CaxWD	1	12637	12637	58.148	***0.0024***	9852	719.38
CaxSD	4	7691.4	1922.8	17.729	***0.0001***	9832	143.7
WDxSD	4	6383.9	1596	14.715	***0.0097***	9826	87.671
Co(CaxWD)	8	17434	2179.2	20.093	***0.0001***	9788	224.54
CaxWDxSD	4	8270.4	2067.6	19.064	***0.0003***	9832	337.04
Res	31	33621	1084.6				1084.6
Total	58	1.30E+05					

PERMANOVA full 4-factor model test for differences between canyon areas (Ca: Gollum and Whittard), water depths (WD: 700 and 1000 m), sediment depths (SD: 1, 2, 3, 4, 5 cm), cores (Co: 1–12), and interaction terms. Bold italic values indicate significant differences at p <0.01. Data was standardised and square root transformed; resemblance was calculated using Bray-Curtis.

The dominant genera for each station are given in Table 2. *Sabatieria* was clearly the dominant genus in the Gollum area, its relative proportions being highest at G1000 (27 and 16% for G1000 and G700, respectively). *Sabatieria* occurred in the Whittard Canyon, but in much lower densities (3.5–4.5%). In contrast, station W1000 was dominated by *Leptolaimus* and *Molgolaimus* (14.4 and 13.5%, respectively), whilst at station W700 mouthless nematodes of the genus *Astomonema* (*A. southwardorum*, containing endo-symbiotic bacteria) constituted more than 10% of the total community.

**Table 2:** Dominance list of nematode genera per station.

G1000	%	G700	%	W1000	%	W700	%
*Sabatieria*	27.0	*Sabatieria*	16.2	*Leptolaimus*	14.4	*Astomonema*	10.3
*Halalaimus*	4.9	*Syringolaimus*	8.2	*Molgolaimus*	13.5	*Halalaimus*	8.9
*Acantholaimus*	4.5	*Halalaimus*	6.8	*Acantholaimus*	9.1	*Acantholaimus*	7.0
*Cervonema*	3.6	*Trefusia*	4.5	*Halalaimus*	5.1	*Sabatieria*	4.5
*Syringolaimus*	3.5	*Acantholaimus*	3.8	*Microlaimus*	5.0	*Terschellingia*	3.9
*Viscosia*	3.0	*Metadesmolaimus*	3.1	*Monhystrella*	4.9	*Metasphaerolaimus*	3.4
*Trefusia*	2.6	*Cervonema*	2.8	*Syringolaimus*	4.5	*Metadesmolaimus*	2.7
*Setosabatieria*	2.3	*Metadasynemella*	2.2	*Desmoscolex*	3.7	aff. *Nannolaimus*	2.3
*Actinonema*	2.3	*Actinonema*	2.1	*Sabatieria*	3.5	*Actinonema*	2.2
*Molgolaimus*	2.0	*Desmodora*	2.0	*Cervonema*	3.2	*Cervonema*	2.0
		*Setosabatieria*	2.0	*Campylaimus*	2.5		

Genera with abundance ≥2%. Mean values presented here based on relative abundances corrected for slice density differences.

The SIMPER test revealed similarity values for the Gollum and Whittard area of 51.5 and 53.8%, respectively (Table 3). As expected from its dominant occurrence at the Gollum stations, *Sabatieria* clearly contributed most to similarity within this area (14.5%). For the Whittard area, a broader range of genera were responsible for similarity between stations. Similarity between the 700 m stations was lower than between the 1000 m stations (49.9 and 55.5%, respectively), and the genus *Sabatieria* contributed most to similarity within these groups. Dissimilarity between the canyon and water-depth groups (57.5–59.4%) was higher than similarity within groups (49.9–55.5%), with *Sabatieria* and *Astomonema* as the main discriminating genera.

**Table 3:** Results from analysis of similarities and species contributions (SIMPER).

**SIMPER similarity (%, contribution >4%)**							
Gollum	51.5	Whittard	53.8	700 m	49.9	1000 m	55.5
*Sabatieria*	14.5	*Sabatieria*	5.5	*Sabatieria*	9.7	*Sabatieria*	10.1
*Trefusia*	5.9	*Syringolaimus*	5.4	*Astomonema*	5.2	*Syringolaimus*	5.9
*Syringolaimus*	5.0	*Monhystrella*	4.8	*Syringolaimus*	4.4	*Microlaimus*	5.5
		*Astomonema*	4.8	*Trefusia*	4.0	*Monhystrella*	5.1
		*Leptolaimus*	4.7			*Leptolaimus*	4.5
		*Microlaimus*	4.5			*Halalaimus*	4.5
		*Halalaimus*	4.5			*Acantholaimus*	4.4
						*Trefusia*	4.3
**SIMPER dissimilarity (%, contribution >3%)**							
Gollum vs. Whittard	59.4	700 m vs. 1000 m	57.5				
*Sabatieria*	4.9	*Astomonema*	4.9				
*Astomonema*	4.6						

Individual genus cut-off level was 4% for similarity analysis, 3% for dissimilarity analysis.

Total nematode biomass was significantly greater in the Gollum Channel than in the Whittard area (Fig. 2, Table 4) with highest values recorded at station G1000 and lowest at W1000 (Fig. 2). No significant differences were observed between water depths. Biomass typically decreased towards the deeper sediment layers.

**Table 4:** Results from univariate PERMANOVA analysis for differences in biomass.

Source	df	SS	MS	Pseudo-F	P(perm)	perms
Ca	1	200.88	200.88	31.914	**0.027**	261
WD	1	13.274	13.274	0.21089	0.605	258
CaxWD	1	30.117	30.117	47.848	0.096	263
Res	4	25.178	62.944			
Total	7	257.5				

PERMANOVA test for biomass data (µg dwt) between canyon areas (Ca: Gollum and Whittard), water depths (WD: 700 and 1000 m), sediment depths (SD: 1, 2, 3, 4, 5 cm), cores (Co: 1–12), and interaction terms. Data was fourth-root transformed; resemblance was calculated using Bray-Curtis. Bold values indicate significant differences at p <0.05.

#### Structural and functional nematode diversity

One hundred and eighty one genera were found among a total of 6051 identified nematodes (*H_0_*, Table 5). The Gollum area was most diverse with 152 genera, compared to 132 genera at the Whittard stations, with 103 genera occurring in both areas. Within the Gollum Channel, only 83 genera were shared between stations, whilst at the Whittard stations, 64 genera were shared. The shallower stations (G700 and W700) harboured more genera (127 and 112, respectively) than the deeper stations G1000 and W1000 (108 and 84 genera, respectively). Generally, Hill's indices indicated that W700 was most diverse and equitable, followed by G700, whilst the deeper stations harboured less diverse and less even communities. At all stations, structural diversity did not show a consistent decrease or increase with sediment depth.

**Table 5:** Structural and functional diversity indices per sediment layer and total per station.

Site	Sediment depth (cm)	TD (Θ^−1^)	*H_0_*	*H_1_*	*H_2_*	*H_inf_*	EG(51)
G700	0–1	3.67	86	67.0	52.3	20.2	39
G700	1–2	3.20	63	43.4	29.5	9.3	24
G700	2–3	3.26	70	53.4	38.8	10.6	34
G700	3–4	3.07	45	37.7	31.2	12.7	45
G700	4–5	2.83	45	36.1	29.2	11.8	33
**G700**	**0–5**	**3.78**	**127**	**46.8**	**20.8**	**6.2**	**27**
G1000	0–1	3.63	70	54.7	44.7	19.5	37
G1000	1–2	2.72	57	39.2	24.4	6.9	26
G1000	2–3	2.62	58	42.5	27.1	7.2	29
G1000	3–4	2.58	49	35.9	25.1	7.6	27
G1000	4–5	3.20	52	38.8	29.3	10.9	25
**G1000**	**0–5**	**3.43**	**108**	**33.5**	**11.5**	**3.7**	**26**
**Gollum**		**3.67**	**152**	**43.8**	**15.9**	**4.6**	**26**
W700	0–1	3.39	73	57.7	45.0	16.0	39
W700	1–2	4.00	84	65.8	52.8	19.9	33
W700	2–3	4.21	62	46.4	33.8	11.5	32
W700	3–4	3.58	42	29.7	20.4	7.4	26
W700	4–5	2.96	37	22.1	13.5	4.9	14
**W700**	**0–5**	**4.37**	**112**	**50.8**	**28.2**	**9.7**	**30**
W1000	0–1	2.78	50	34.9	25.4	10.9	27
W1000	1–2	3.12	56	40.1	29.8	12.1	27
W1000	2–3	3.57	43	30.7	23.1	10.7	25
W1000	3–4	3.70	40	33.0	28.2	12.8	30
W1000	4–5	3.29	35	28.4	23.8	12.2	31
**W1000**	**0–5**	**3.10**	**84**	**27.6**	**15.7**	**6.9**	**21**
**Whittard**		**3.75**	**132**	**48.7**	**28.3**	**12.4**	**29**
**Gollum-Whittard**		**3.86**	**181**	**53.9**	**26.8**	**7.8**	**29**

Community abundance data was averaged over replicates. For total values, relative abundances were first corrected for density differences per sediment layer.

Trophic diversity (TD) is shown in Table 5. Trophic diversity was higher at the 700 m stations than at the 1000 m stations, and highest at W700 (4.37). This was due to relatively high numbers of the gutless nematodes belonging to the genus *Astomonema* at this station. Lowest TD was observed at station W1000 (3.10). At the Gollum stations, the non-selective deposit feeders (1B) dominated in terms of relative abundance, whilst at the Whittard stations the selective deposit feeders (1A) dominated the assemblage. At all stations biomass was dominated by the non-selective deposit feeders (1B). Striking was the increasing numbers of *Astomonema* nematodes with increasing sediment depth at station W700. This was also the case, but to a much lesser extent, at G1000, where non-selective deposit feeders (1B) were dominant.

#### Relation between structure and function of nematodes and environmental variables

**Figure 5:**
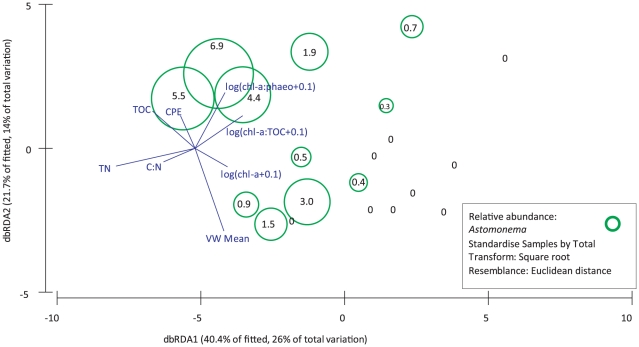
Distance-based redundancy (dbRDA) bubble plot illustrating the DISTLM model based on the genera assemblage data and fitted environmental variables with their vector (strength and direction of effect of the variable on the ordination plot). Axis legends include % of variation explained by the fitted model and % of total variation explained by the axis. Relative abundance of the ‘chemosynthetic’ *Astomonema* nematodes is visualised by the size of the circles, including the value of their relative abundance in each sample. Chl-a: chlorophyll a, CPE: chloroplastic pigment equivalents, Chl-a∶phaeo: chlorophyll a divided by its degradation products (phaeophytines) indicating ‘freshness’ of the phytodetrital OM, TN: total nitrogen content, TOC: total organic carbon content, C∶N: molar carbon-nitrogen ratio, Chl-a∶TOC: chlorophyll a divided by total organic carbon content indicating bioavailability of the bulk OM, CPE∶TOC: total pigment derived matter relative to the bulk OM, VW Mean: volume weighted mean grain size.

The RELATE analysis indicated that the spatial pattern based on the environmental variables is significantly related to the patterns inherent to the community structure (*ρ* = 0.27, p<0.01). Of the original set of 18 environmental parameters, 9 were retained for further analysis based on collinearity analysis (Draftsman plot, Fig. S1). Nine variables with correlation r^2^ values >0.9 (considered redundant) were omitted for the DISTLM procedures; the remaining variables and their pair-wise spearman correlations are shown in Table S4. The best fitted model, based on the retained environmental variables, that explains the genera assemblage is shown in Table S5 and illustrated in Fig. 5. These results show that availability of OM (chl-a∶TOC) explains nearly 17% of the variation observed. Other main contributors in explaining nematode genera assemblage variation in genera assemblages between samples include TN (14.2%), mean grain size (11.3%) and chl-a content (6.4%) of the sediments. Other variables contributed <5% in explaining assemblage variation. Of the 9 variables, 8 were used by the DISTLM procedure to construct the best-fitting model, together explaining 64.3% of total variation. The DISTLM results for the univariate biotic parameters are summarised in Table S6. They reveal that the relative abundance and biomass of *Astomonema* are mainly explained by TOC and TN in the sediments, while TD variability is mainly explained by TN. Nematode density and diversity indices are controlled by chl-a-derived parameters, indicating the important role of the quality and quantity of phytodetrital OM for these biotic parameters. Fig. 5 shows the DISTLM results by means of a dbRDA plot, with the relative abundance of *Astomonema* superimposed. The vectors of the environmental variables retained by the DISTLM procedure as fitting the best explanatory model indicate the important role of TOC, TN and C∶N in explaining their abundance.

Kendall-Tau correlations between structural community parameters and environmental variables are shown in Table 6. There were differences between the Gollum and Whittard stations, with 34 and 21 significant correlations, respectively, indicating different levels of interactions between the two areas.

**Table 6:** Kendall-Tau correlation coefficients between biotic and abiotic variables.

Parameter	Chl-a	CPE	Chl-a∶phaeo	TN	TOC	C∶N	Chl-a∶TOC	CPE∶TOC	Clay %	Silt %	Sand %
**Gollum**											
NemaDens	***0.653***	***0.835***	**0.544**	**0.600**	0.378	0.244	**0.599**	***0.740***	0.333	**0.511**	**−0.556**
Chemos. RA	−0.216	−0.162	−0.339	0.479	**0.630**	**0.580**	−0.278	−0.271	***0.731***	0.428	−0.378
NemaBiom	**0.544**	***0.692***	**0.544**	0.467	0.244	0.111	**0.490**	***0.692***	0.289	0.467	**−0.511**
Chemos. Biomass	0.031	0.054	−0.093	**0.580**	***0.731***	**0.580**	−0.031	−0.054	**0.630**	**0.630**	**−0.580**
TD	0.435	0.358	0.435	−0.067	0.067	0.022	**0.490**	0.454	−0.156	−0.067	0.022
*H_0_*	**0.585**	**0.635**	**0.585**	0.159	0.114	0.159	***0.640***	***0.733***	0.068	0.159	−0.205
*H_1_*	**0.599**	***0.645***	**0.599**	0.111	0.067	0.111	***0.653***	***0.740***	0.022	0.111	−0.156
EG(51)	0.109	0.072	0.000	−0.200	−0.244	−0.022	0.054	0.072	−0.378	−0.200	0.156
**Whittard**											
NemaDens	0.368	0.405	**0.529**	0.200	−0.156	−0.244	**0.552**	**0.494**	***−0.644***	−0.022	0.022
Chemos. RA	0.420	0.290	0.162	0.406	***0.835***	***0.788***	0.222	0.193	0.215	**0.501**	**−0.501**
NemaBiom	**0.506**	**0.539**	**0.529**	0.378	0.111	0.022	**0.506**	**0.539**	−0.467	0.244	−0.244
Chemos. Biomass	0.469	0.338	0.217	0.358	***0.788***	***0.740***	0.272	0.145	0.263	0.454	**−0.549**
TD	−0.046	0.090	0.227	0.200	0.289	0.378	−0.230	−0.090	0.244	0.422	−0.422
*H_0_*	0.460	**0.494**	**0.580**	0.289	0.022	−0.067	0.368	0.405	−0.378	0.244	−0.244
*H_1_*	0.368	0.405	**0.529**	0.244	−0.022	−0.111	0.276	0.315	−0.244	0.200	−0.200
EG(51)	0.256	0.386	**0.510**	**0.494**	0.135	0.045	0.163	0.295	0.135	0.405	−0.405

Chl-a: chlorophyll a, CPE: chloroplastic pigment equivalents, Chl-a:phaeo: chlorophyll a divided by its degradation products (phaeophytines) indicating ‘freshness’ of the phytodetrital OM, TN: total nitrogen content, TOC: total organic carbon content, C∶N: molar carbon-nitrogen ratio, Chl-a∶TOC: chlorophyll a divided by total organic carbon content indicating bioavailability of the bulk OM, CPE∶TOC: total pigment derived matter relative to the bulk OM. NemaDens: nematode abundance, Chemos. RA: relative abundance of the chemosynthetic *Astomonema* nematodes, NemaBiom: total nematode biomass, Chemos. Biomass: total biomass of the chemosynthetic *Astomonema* nematodes. Bold values denote significance at the p<0.05 level. Bold, italic values denote significance at the p <0.01 level.

In the Gollum area, nematode density, nematode biomass, *H_0_*, and *H_1_* were positively correlated with the quality and bioavailability of OM (Chl-a, CPE, ‘freshness’ (Chl-a∶phaeo), bioavailability (Chl-a∶TOC). Nematode density was positively correlated with TN and silt content and showed a negative relation with sand content. The relative abundance and biomass of *Astomonema* showed no correlation with chl-a and its derivatives. However, the biomass of this genus was positively correlated with TOC, TN, and C∶N while its relative abundance was positively correlated with TOC and C∶N. Total *Astomonema* biomass was greater in finer sediments than in coarser sediments as inferred from the correlations with grain-size fractions. Trophic diversity was positively correlated with chl-a∶TOC. Genus diversity (*H_0_*, *H_1_*) was positively correlated with chl-a and chl-a-derived variables.

In the Whittard area, nematode density was positively correlated with ‘freshness’ and availability of OM (chl-a∶phaeo, chl-a∶TOC), whilst a negative relation was found with clay content. *Astomonema* relative abundance and biomass was positively correlated with TOC and C∶N and negatively correlated with sand content. Nematode biomass was positively correlated with chl-a and all chl-a-derived variables, indicating its dependence on photosynthetically derived material. *H_0_*, *H_1_*, and EG(51) were positively related to ‘freshness’ values (Chl-a∶phaeo).

## Discussion

### Quantity and quality of organic matter and its effect on meiofauna abundance and biomass

At the Celtic Margin area, the high productivity in surface waters leads to significant sedimentation of OM to the deep sea [53], [54], which may positively control meiofauna standing stocks [55], [56], [57], [58], [59], [60]. Meiofauna abundances and biomass were higher (1054–1426 ind. 10 cm^−2^, 89.2–276.8 µg dwt) in the Whittard and Gollum area compared to values reported for areas such as the Iberian Margin or the Mediterranean Sea at similar depths (see Soltwedel [57] for review), but similar to values observed previously along the Celtic Margin [58], [61]. In contrast, at the Goban Spur, in between the Gollum and Whittard area, Vanaverbeke et al. [62] found meiofauna densities of 612 and 619 ind. 10 cm^−2^ at 670 and 1034 m depth respectively and suggested that lower OM input was responsible for the lower densities compared to neighbouring areas that receive higher amounts of phytodetritus. On a global scale, the Gollum and Whittard stations exhibit much greater meiobenthic standing stocks when compared to trenches/canyons and similar depth zones [60]. Enhanced accumulation of OM in topographically enclosed areas or canyon/channel structures is a well-known phenomenon [5], [6], [60], [63], [64], and is often seen to result in benthic abundance and biomass hotspots [11], [60], [65], [66], [67]. For the Gollum and Whittard area, the positive control of food sources on meiofauna standing stocks is illustrated by the strong correlations of nematode densities and biomass with the quality and bioavailability of the OM (Table 6) and the high contributions of freshness (chl-a∶phaeo) and CPE∶TOC in explaining variation of total nematode densities and biomass (Table S6). However, the correlations were more frequent and stronger in the Gollum Channel where currents are capable of producing significant turbidity compared to the Whittard study area, where hydrodynamic disturbance is less intense and less frequent [26], [33], [35]. This suggests that the relation between nematode abundance and biomass, and quantity, quality and bioavailability of the present food is stronger when hydrodynamic disturbance is greater, providing that the disturbance is not of such proportions that it is able to preclude the establishment of meiobenthic communities. Interestingly, Bianchelli et al. [12] suggested for the Bari Canyon (Adriatic Sea) that the trophic relations in canyon sediments are tightly connected with the hydrodynamic conditions and that such a relation becomes tighter as the hydrodynamic forcing increases. Even though this hypothesis needs to be substantiated by more observations from different canyon and channel systems, it corroborates the Dynamic Equilibrium Model and there are other indications for its validity. Disturbance of deep-sea, soft-bottom communities at an intermediate level, i.e. when not detrimental to the sustenance of the community, can explain high benthic standing stocks because of the replenishment of available food sources through cycles of resuspension and deposition of OM [68]. Higher rates of hydrodynamic disturbance may therefore enhance food availability and intensify the positive relation between food sources in the sediment and benthic standing stocks. Another possible explanation for the high meiofauna numbers and biomass at the Gollum and Whittard stations may be the warming influence of the Mediterranean Outflow Water (MOW, [33]) and its positive effect on bacterial biomass production. Since many deep-sea nematodes are specific bacteria feeders or general microbial feeders, enhanced bacterial biomass under the influence of the warmer and more saline MOW compared to other locations at similar depths, may result in an increase of suitable organic food sources and lead to greater standing stocks [69].

The C∶N ratio can give an indication of whether the sedimentary OM is derived from a terrestrial source or whether it is of a marine origin. However, preferential removal of labile, proteinaceous (i.e. N-rich) marine OM during degradation processes may also lead to higher C∶N ratios. At station G1000 and W700, C∶N values were very high, indicating the presence of either large amounts of terrigenous material or highly degraded OM. Our sampling sites are not located near a river system from which they could receive substantial fluvial export, as is the case for other canyon systems such as the Nazaré and Setúbal canyons at the Portuguese Margin. It is therefore very likely that enhanced degradation of the OM at these stations is responsible for the high C∶N ratios observed. Enhanced aerobic decay of high organic loads may lead to reducing conditions and limited oxygen availability, and ultimately lower infauna abundances and biomass beyond expected for the observed food input [23], [70], [71], but also change the composition of the resident fauna. This is illustrated by the biogeochemical conditions at station W700. Even though sediments at W700 displayed greatest levels of TOC and CPE and highest chl-a∶phaeo and chl-a∶TOC values (Fig. 2, 3), meiofauna densities and nematode biomass were not higher compared to the other stations (Fig. 2), possibly due to oxygen limitation in the sediment. Further arguments for this are given in the discussion of the nematode community assemblage of station W700.

Currents preferentially transport finer grained sediments, causing the remaining sediment to become relatively coarser [72]. This is in agreement with coarser sediments being present at the Gollum stations compared to the Whittard stations when considering current activity in these areas, and reflects the Gollum Channel's role as preferential conduit for the transport of sediment from shelf depths to the deep sea. Since currents in the Gollum Channels are able to transport the lighter sediment fractions down canyon, the upper reaches of this channel system will be characterised by the coarser remaining fraction, as illustrated by the grain size differences between G700 and G1000 (Fig. 3). For the Gollum stations, nematode densities and biomass were negatively correlated with sand content of the sediments. However, we expect that this is not purely a causal relation but rather a result of a decrease in grain size with sediment depth coinciding with decreasing nematode abundance and biomass.

### Nematode community structure and function, and their relation with environmental conditions

The generic assemblages were significantly different between the Gollum and Whittard stations, between 700 and 1000 m water depth, and between individual stations (Table 1). Furthermore, nematode composition differed significantly between different sediment layers (Table 1), which was the most important factor across canyons and water depths as indicated by the estimated components of variation (Table 1) and illustrated in the MDS plot (Fig. 4). The contrasting community differences between sediment layers suggest that biogeochemical gradients in the sediment have a greater impact on nematode community structure than gradients related to canyon area, water depth, and station differences. Moreover, assemblage differences in the deeper sediment layers were more pronounced than at the surface, a pattern that was also observed for the Nazaré, Cascais and Setúbal canyons and the open slope at the Portuguese Margin [11], [66]. The prevailing effect of sediment depth is most probably related to the differential ability of different genera to reside in or penetrate different sediment layers and has been associated with oxygen availability in the sediments [23], [70]. Striking in this respect is the occurrence of high numbers of *Astomonema southwardorum* at station W700 and G1000, especially in the deeper sediment layers. Nematodes of the genus *Astomonema* have a degenerated alimentary canal; they posses no mouth, no buccal cavity, nor pharynx, and they have a rudimentary gut containing micro-organisms [73], [74], [75], [76]. Until now, *A. southwardorum* had only been found in a methane seep pockmark in the North Sea [73]. Mouthless and gutless nematodes carrying endosymbionts have been observed previously in near-anoxic, deep-sea environments rich in OM [77], but this is the first time that *Astomonema* has been recovered from the deep-sea. The genus *Astomonema* is morphologically closely related to the genus *Parastomonema*, and has been classified in the same subfamily Astomonematinae [78]. The prokaryotic endosymbionts associated with *A. southwardorum* are now recognised to be sulphur-oxidising chemolithoautotrophs, which reduce sulphur compounds as an energy source to provide their nematode hosts with nutrition [74], [75], [79]. The abundance of *Astomonema* was positively correlated with TOC, TN, and C∶N values and the variation in its abundance and biomass was mainly explained by TOC and TN content in the sediments (Table S6), suggesting that they are directly or indirectly dependent on the quantity and level of decomposition of OM. No correlations with any chl-a derived variable was observed (Table 6), nor were there important contributions of chl-a derived variables in explaining its abundance and biomass variability (Table S6), indicating their independence of fresh food sources. Enhanced aerobic respiration of great amounts of OM may lead to reducing conditions whereby anoxic mineralisation leads to reduced byproducts such as sulphides. The presence of the ‘chemosynthetic’ *Astomonema* at W700 and G1000, where OM levels and C∶N ratios (as a measure of OM degradation) are greatest, supports the idea of a sulphidic biome in reducing sediment conditions being present at these stations. This is also attested by higher *Astomonema* abundances in deeper sediment layers, where oxygen availability is likely lower and the concentration of sulphidic compounds higher than at the surface. Such environments constitute ideal habitats for *Astomonema* nematodes to thrive in, as their endosymbiotic, thiotrophic bacteria may provide them with a competitive advantage over other feeding types. Moreover, their filiform shape is characteristic for nematodes in oxygen-limited environments and may result in increased mobility, which enables these organisms to migrate between anoxic patches and parts of the sediment where oxygen is available and/or to bridge the gap between oxic and anoxic patches with their body [23]. The presence of the ‘chemosynthetic’ *Astomonema* at stations with high organic input is in agreement with the TROX model [22] in the sense that in eutrophic systems, the faunal community seems oxygen driven as opposed to a food-driven community in oligotrophic conditions. *Astomonema* is able to exploit the reduced environment where patchy oxygen limitation hinders the survival of other, less tolerant genera and can use reduced compounds as a food resource through an endosymbiotic pathway. A complementary explanation for the prevalence of *Astomonema* in deeper sediment layers could be that in deeper layers they would be better protected against possible predators. Interestingly, many of the *Astomonema* individuals exhibited different stages of wound healing at their posterior end, an indication that these nematodes are being preyed on, but have the ability to escape their attackers. Possibly, their long, flexible bodies enable them to avoid being killed through hind-body loss which may be facilitated by cuticle structure in order to survive predation (Tchesunov, unpublished data). The same phenomenon of hind-body wound healing is found with typically very long *Siphonolaimus* individuals from the White Sea (Tchesunov, unpublished data).

Dominant genera differed greatly between stations (Table 2). Whilst *Sabatieria* consistently dominated at the Gollum stations, the nematode assemblage at W700 was controlled by the genus *Astomonema* and at W1000 by the genera *Leptolaimus* and *Molgolaimus* (Table 2). Many *Sabatieria* species are adapted to exploit newly available habitats or resources [13], [80] and are typically found in unpredictable and variable environments [38], [81]. The dominance of *Sabatieria* at the disturbed and hydrodynamically active Gollum stations is thus not surprising. In contrast, *Leptolaimus* and *Molgolaimus*, the dominant genera at W1000, are usually not regarded as opportunistic but rather as persisters (cf. definitions *sensu* Bongers et al. [82]) and are not considered able to endure intense sediment disturbance [80]. This suggests that sediments at this station are much more stable and less disturbed compared to those at the Gollum stations, which is confirmed by our knowledge on hydrodynamic activity in these areas [33]. On the other hand, ex-situ colonisation experiments investigating the ability of nematodes to actively colonise defaunated sediments revealed that *Leptolaimus* nematodes may be considered active colonisers [83]. However, the *Sabatieria* and *Leptolaimus* species found to colonise the defaunated sediments [83] often occur in deeper, hypoxic to anoxic sediment layers [84]. Moreover, the defaunated sediments used for the experiment originated from deeper sediment layers (>8 cm depth) and biogeochemical characteristics such as oxygen availability may have been different compared to natural surface and subsurface sediments. Hence, the preferential migration of *Leptolaimus* to the defaunated sediments may have been triggered by the biogeochemical properties of the substratum used.

Genus richness (*H_0_*) at all four stations (84–127 genera) was very high in comparison with reported values by Vanaverbeke et al. [62] for similar depths at the Goban Spur (45–60). Even when comparing *H_0_* from the present study with values from a cold-water coral habitat in the Porcupine Seabight [85], which is globally considered one of the most diverse nematode habitats [86], genus richness is still moderately higher at the Gollum and Whittard stations (91–106 and 84–127 for the cold-water coral habitat and the present study, respectively). For the Gollum area strong positive correlations were found between *H_0_*, *H_1_* and chl-a, CPE, chl-a∶phaeo, and chl-a∶TOC, indicating that structural diversity is related to the quality and bioavailability of the OM present (Table 6). In contrast, correlations in the Whittard area were much less pronounced or not significant. This suggests that the underlying causes for structural nematode diversity have a differential effect in these two study areas. More importantly, it indicates that diversity in the Gollum Channels may be more dependent on the trophic conditions (i.e. quantity, quality, and bioavailability of the OM) than is the case for the Whittard area. This difference may reflect the differential hydrodynamic regimes in the two areas. It suggests that increased hydrodynamic activity, as in the Gollum Channels, enhances the relation between structural diversity and food quality and availability, as was the case for nematode abundance and biomass. From this perspective, the intermediate disturbance hypothesis, which is generally applicable to deep-sea communities [68], can be evoked. An intermediate disturbance frequency may promote high diversity, providing there is enough time between disturbance events for species to colonise and establish themselves in the new habitats. Too much time between disturbance events, and competitive interactions and exclusion may cause diversity to decline. Combined with enhanced food availability because of cycles of resuspension and deposition caused by recurring hydrodynamic disturbance, these factors may explain the tighter positive relation between nematode structural diversity and trophic conditions in sediments at the Gollum stations compared to the Whittard Canyon.

Nematode trophic diversity (TD) was correlated with chl-a∶TOC at the Gollum stations and this was not the case for the Whittard stations. This may imply that TD is differentially influenced by trophic conditions when comparing both areas. However, the lack of correlations between TD and other trophic parameters suggests that, if such a contrast is even present, it occurs to a much lesser extent. Both structural and trophic diversity were lower at the deeper stations than at the shallower stations within each study area, and both seemed to be controlled to some extent by trophic conditions (see DISTLM results, Table S6), something which is also supported by the observation that the quality of food at the deeper stations is lower than at the shallower stations (Fig. 2, 3). On the other hand, the high amounts and freshness of the OM, and the prevalence of *Astomonema* at W700 (resulting in a high TD and indicative of the use of reduced compounds as food source) agree with the principle of more trophic levels being present when food input is greater [87]. This is also confirmed by higher TD at sites with higher organic input caused by greater relative abundances and biomass of predators/scavengers [11]. With this in mind, it can be argued that the ecofunctional importance of *Astomonema* in the community is transferring the energy contained in available sulphur compounds into nematode biomass so that the high trophic complexity of the community is sustained by providing a food source to nematode predators/scavengers and macrofauna. The presence of *Astomonema* as a result of local trophic conditions indicates the importance of small-scale heterogeneity and biogeochemical patterns along the vertical sediment profile for structural and trophic diversity in canyon environments.

## Supporting Information

Figure S1 **Draftsman plot or scatter plot matrix of environmental variables to assess collinearity between environmental variables.** Chl-a: chlorophyll a, CPE: chloroplastic pigment equivalents, Chl-a∶phaeo: chlorophyll a divided by its degradation products (phaeophytines) indicating ‘freshness’ of the phytodetrital OM, TN: total nitrogen content, TOC: total organic carbon content, C∶N: molar carbon-nitrogen ratio, Chl-a∶TOC: chlorophyll a divided by total organic carbon content indicating bioavailability of the bulk OM, CPE∶TOC: total pigment derived matter relative to the bulk OM, VW Mean: volume weighted mean grain size.
(EPS)

Table S1 **Results from univariate PERMANOVA analyses for differences in sedimentary abiotic variables.** PERMANOVA test for canyon areas (Ca: Gollum and Whittard), water depths (WD: 700 and 1000 m), sediment depths (SD: 1, 2, 3, 4, 5 cm), cores (Co: 1–12), and interaction terms. Chl-a: chlorophyll a, CPE: chloroplastic pigment equivalents, Chl-a∶phaeo: chlorophyll a divided by its degradation products (phaeopigments) indicating ‘freshness’ of the phytodetrital OM, Chl-a∶TOC: chlorophyll a divided by total organic carbon content indicating bioavailability of the bulk OM; TOC: total organic carbon, TN: total nitrogen, C∶N: molar carbon/nitrogen ratio, Mean grain size: volume weighted grain size. Data was normalised; resemblance was calculated using Euclidean Distance. Bold values indicate significant differences at p <0.05, bold italic values indicate significant differences at p <0.01.
(DOCX)

Table S2 **Meiofauna taxa abundances per sediment layer.** Values denote means of replicates (ind. 10 cm^−2^). St. Dev.: standard deviation. G1000: Gollum Channel at 1090 m depth, G700: Gollum Channel at 755 m depth, W1000: Whittard Canyon at 1160 m depth, W700: Whittard Canyon at 762 m depth.
(DOCX)

Table S3 **Results** **from pair-wise multivariate PERMANOVA analyses for differences in nematode community structure.** PERMANOVA pair-wise comparisons within each of the Ca×WD×SD levels as part of the repeated measures analysis, including Monte-Carlo permutation p values (P(MC)). The pair-wise comparisons give an indication of the individual contribution to the significant three-way interaction term (Ca×WD×SD) with differences between canyon areas (Ca: Gollum and Whittard), water depths (WD: 700 and 1000 m), sediment depths (SD: 1, 2, 3, 4, 5 cm). Data was standardised and square root transformed; resemblance was calculated using Bray-Curtis. Bold values indicate significant differences at p <0.05.
(DOCX)

Table S4 **Spearman correlation coefficients between environmental variables.** Chl-a: chlorophyll a, CPE: chloroplastic pigment equivalents, Chl-a∶phaeo: chlorophyll a divided by its degradation products (phaeophytines) indicating ‘freshness’ of the phytodetrital OM, TN: total nitrogen content, TOC: total organic carbon content, C∶N: molar carbon-nitrogen ratio, Chl-a∶TOC: chlorophyll a divided by total organic carbon content indicating bioavailability of the bulk OM, CPE∶TOC: total pigment derived matter relative to the bulk OM.
(DOCX)

Table S5 **Distance-based linear model (DISTLM) for genera assemblages and selected environmental variables.** Variables: Selected environmental variables used to calculate the optimum model. Marginal tests: explanation of variation for each variable taken alone. Sequential tests: conditional tests of individual variables in constructing the model. Each test examines whether adding the variable contributes significantly to the explained variation. Selection procedure: step-wise, selection criterion: adjusted R^2^. Prop.: % variation explained. Cumul.: cumulative variation explained. Chl-a: chlorophyll a, CPE: chloroplastic pigment equivalents, Chl-a∶phaeo: chlorophyll a divided by its degradation products (phaeophytines) indicating ‘freshness’ of the phytodetrital OM, TN: total nitrogen content, TOC: total organic carbon content, C∶N: molar carbon-nitrogen ratio, Chl-a∶TOC: chlorophyll a divided by total organic carbon content indicating bioavailability of the bulk OM, CPE∶TOC: total pigment derived matter relative to the bulk OM, Mean grain size: volume weighted mean grain size.
(DOCX)

Table S6 **Distance-based linear models (DISTLM) - Sequential tests for best fitting model for each of the univariate biotic parameters.** Sequential tests: conditional tests of individual variables in constructing the model. Each test examines whether adding the variable contributes significantly to the explained variation. Selection procedure: step-wise, selection criterion: adjusted R^2^. Prop.: % variation explained. Cumul.: cumulative variation explained. NemaDens: nematode abundance, Chemos. RA: relative abundance of the chemosynthetic *Astomonema* nematodes, NemaBiom: total nematode biomass, Chemos. Biomass: total biomass of the chemosynthetic *Astomonema* nematodes, TD: trophic diversity, *H_0_*: genus richness, *H_1_*: Hill's diversity index, EG(51): expected number of genera. Chl-a: chlorophyll a, CPE: chloroplastic pigment equivalents, Chl-a∶phaeo: chlorophyll a divided by its degradation products (phaeophytines) indicating ‘freshness’ of the phytodetrital OM, TN: total nitrogen content, TOC: total organic carbon content, C∶N: molar carbon-nitrogen ratio, Chl-a∶TOC: chlorophyll a divided by total organic carbon content indicating bioavailability of the bulk OM, CPE∶TOC: total pigment derived matter relative to the bulk OM, Mean grain size: volume weighted mean grain size.
(DOCX)
