# Supplementary material for: Meiofauna in the Gollum Channels and the Whittard Canyon, Celtic Margin—How Local Environmental Conditions Shape Nematode Structure and Function
Source: PLoS One. 2011 May 18;6(5):e20094. doi: 10.1371/journal.pone.0020094 (PMC3097227; doi:10.1371/journal.pone.0020094)
Supplement: Table S1 — Results from univariate PERMANOVA analyses for differences in sedimentary abiotic variables. PERMANOVA test for canyon areas (Ca: Gollum and Whittard), water depths (WD: 700 and 1000 m), sediment depths (SD: 1, 2, 3, 4, 5 cm), cores (Co: 1–12), and interaction terms. Chl-a: chlorophyll a, CPE: chloroplastic pigment equivalents, Chl-a∶phaeo: chlorophyll a divided by its degradation products (phaeopigments) indicating ‘freshness’ of the phytodetrital OM, Chl-a∶TOC: chlorophyll a divided by total organic carbon content indicating bioavailability of the bulk OM; TOC: total organic carbon, TN: total nitrogen, C∶N: molar carbon/nitrogen ratio, Mean grain size: volume weighted grain size. Data was normalised; resemblance was calculated using Euclidean Distance. Bold values indicate significant differences at p <0.05, bold italic values indicate significant differences at p <0.01. (DOCX) [file pone.0020094.s002.docx]

**Table S1.** Results from univariate PERMANOVA analyses for differences in sedimentary abiotic variables

| **Chl-a** | df | SS | MS | Pseudo-F | P(perm) | perms |  | **TOC** | df | SS | MS | Pseudo-F | P(perm) | perms |
| --- | --- | --- | --- | --- | --- | --- | --- | --- | --- | --- | --- | --- | --- | --- |
| Ca | 1 | 14.471 | 14.471 | 10.83 | **0.0203** | 9743 |  | Ca | 1 | 0.55443 | 0.55443 | 0.47469 | 0.4965 | 9774 |
| WD | 1 | 36.522 | 36.522 | 27.334 | ***0.0045*** | 9766 |  | WD | 1 | 0.55443 | 0.55443 | 0.47469 | 0.4944 | 9782 |
| SD | 4 | 11.523 | 28.807 | 33.315 | **0.031** | 9954 |  | SD | 4 | 27.569 | 0.68923 | 10.658 | 0.3971 | 9948 |
| CaxWD | 1 | 25.658 | 25.658 | 19.203 | ***0.0045*** | 9770 |  | CaxWD | 1 | 98.249 | 98.249 | 84.118 | **0.0277** | 9733 |
| CaxSD | 4 | 0.49131 | 0.12283 | 0.14205 | 0.963 | 9954 |  | CaxSD | 4 | 25.521 | 0.63802 | 0.98662 | 0.4351 | 9948 |
| WDxSD | 4 | 29.869 | 0.74673 | 0.86358 | 0.5039 | 9958 |  | WDxSD | 4 | 29.488 | 0.7372 | 1.14 | 0.3605 | 9953 |
| co(CaxWD) | 7 | 0.44793 | 0.0640 | 0.0740 | 0.9994 | 9932 |  | co(CaxWD) | 7 | 85.234 | 12.176 | 18.829 | 0.1162 | 9948 |
| CaxWDxSD | 4 | 0.59401 | 0.1485 | 0.17174 | 0.9505 | 9950 |  | CaxWDxSD | 4 | 23.225 | 0.58062 | 0.89786 | 0.4786 | 9956 |
| Res | 23 | 19.888 | 0.86469 |  |  |  |  | Res | 23 | 14.874 | 0.64668 |  |  |  |
| Total | 49 | 49 |  |  |  |  |  | Total | 49 | 49 |  |  |  |  |
|  |  |  |  |  |  |  |  |  |  |  |  |  |  |  |
| **CPE** | df | SS | MS | Pseudo-F | P(perm) | perms |  | **TN** | df | SS | MS | Pseudo-F | P(perm) | perms |
| Ca | 1 | 35.703 | 35.703 | 45.829 | 0.0691 | 9783 |  | Ca | 1 | 33.822 | 33.822 | 83.235 | **0.0269** | 9782 |
| WD | 1 | 47.948 | 47.948 | 61.548 | **0.0488** | 9741 |  | WD | 1 | 70.097 | 70.097 | 17.251 | **0.0103** | 9707 |
| SD | 4 | 95.744 | 23.936 | 88.122 | ***0.0004*** | 9956 |  | SD | 4 | 0.78261 | 0.19565 | 24.703 | **0.0719** | 9968 |
| CaxWD | 1 | 8.531 | 8.531 | 10.951 | **0.0147** | 9766 |  | CaxWD | 1 | 22.579 | 22.579 | 55.566 | ***0.0028*** | 9752 |
| CaxSD | 4 | 0.0567 | 0.0142 | 0.0521 | 0.9943 | 9944 |  | CaxSD | 4 | 0.24312 | 0.0608 | 0.76738 | 0.5611 | 9957 |
| WDxSD | 4 | 0.0999 | 0.0250 | 0.0919 | 0.981 | 9966 |  | WDxSD | 4 | 0.21889 | 0.0547 | 0.69091 | 0.608 | 9945 |
| co(CaxWD) | 7 | 57.916 | 0.82737 | 3.046 | **0.0185** | 9958 |  | co(CaxWD) | 7 | 30.625 | 0.4375 | 55.238 | ***0.0013*** | 9945 |
| CaxWDxSD | 4 | 15.328 | 0.38319 | 14.107 | 0.2593 | 9952 |  | CaxWDxSD | 4 | 0.34681 | 0.0867 | 10.947 | 0.3849 | 9954 |
| Res | 23 | 62.473 | 0.27162 |  |  |  |  | Res | 23 | 18.217 | 0.0792 |  |  |  |
| Total | 49 | 49 |  |  |  |  |  | Total | 49 | 49 |  |  |  |  |
|  |  |  |  |  |  |  |  |  |  |  |  |  |  |  |
| **Chl-a:Phaeo** | df | SS | MS | Pseudo-F | P(perm) | perms |  | **C:N** | df | SS | MS | Pseudo-F | P(perm) | perms |
| Ca | 1 | 0.52458 | 0.52458 | 0.71222 | 0.4323 | 9809 |  | Ca | 1 | 0.94671 | 0.94671 | 0.92384 | 0.4066 | 9820 |
| WD | 1 | 2.279 | 2.279 | 30.942 | 0.1206 | 9770 |  | WD | 1 | 0.0220 | 0.0220 | 0.0215 | 0.9086 | 9815 |
| SD | 4 | 16.753 | 41.882 | 87.092 | ***0.0005*** | 9949 |  | SD | 4 | 16.545 | 0.41363 | 0.41336 | 0.7963 | 9961 |
| CaxWD | 1 | 0.44546 | 0.44546 | 0.6048 | 0.5123 | 9658 |  | CaxWD | 1 | 8.379 | 8.379 | 81.766 | **0.0281** | 9784 |
| CaxSD | 4 | 50.144 | 12.536 | 26.068 | 0.0586 | 9948 |  | CaxSD | 4 | 36.371 | 0.90927 | 0.90869 | 0.4674 | 9956 |
| WDxSD | 4 | 0.85269 | 0.21317 | 0.44328 | 0.7795 | 9951 |  | WDxSD | 4 | 0.63465 | 0.15866 | 0.15856 | 0.9593 | 9951 |
| co(CaxWD) | 7 | 53.263 | 0.7609 | 15.822 | 0.1909 | 9940 |  | co(CaxWD) | 7 | 71.894 | 10.271 | 10.264 | 0.4408 | 9948 |
| CaxWDxSD | 4 | 18.518 | 0.46295 | 0.96269 | 0.4523 | 9938 |  | CaxWDxSD | 4 | 1.394 | 0.34849 | 0.34826 | 0.8381 | 9948 |
| Res | 23 | 11.061 | 0.4809 |  |  |  |  | Res | 23 | 23.015 | 10.006 |  |  |  |
| Total | 49 | 49 |  |  |  |  |  | Total | 49 | 49 |  |  |  |  |
|  |  |  |  |  |  |  |  |  |  |  |  |  |  |  |
| **Chl-a:TOC** | df | SS | MS | Pseudo-F | P(perm) | perms |  | **Mean grain size** | df | SS | MS | Pseudo-F | P(perm) | perms |
| Ca | 1 | 0.64822 | 0.64822 | 0.87646 | 0.4064 | 9703 |  | Ca | 1 | 20.127 | 20.127 | 36.884 | ***0.0023*** | 9757 |
| WD | 1 | 2.853 | 2.853 | 38.575 | 0.0893 | 9793 |  | WD | 1 | 0.69648 | 0.69648 | 12.764 | 0.2979 | 9777 |
| SD | 4 | 18.085 | 45.212 | 79.374 | ***0.0003*** | 9962 |  | SD | 4 | 0.0927 | 0.0232 | 0.60602 | 0.6597 | 9962 |
| CaxWD | 1 | 0.0198 | 0.0198 | 0.0268 | 0.8785 | 9767 |  | CaxWD | 1 | 19.971 | 19.971 | 36.598 | ***0.0024*** | 9783 |
| CaxSD | 4 | 0.37409 | 0.0935 | 0.16419 | 0.9558 | 9956 |  | CaxSD | 4 | 0.73835 | 0.18459 | 48.247 | ***0.0052*** | 9957 |
| WDxSD | 4 | 53.052 | 13.263 | 23.285 | 0.0854 | 9964 |  | WDxSD | 4 | 0.91376 | 0.22844 | 5.971 | ***0.002*** | 9954 |
| co(CaxWD) | 7 | 52.905 | 0.75578 | 13.268 | 0.2888 | 9954 |  | co(CaxWD) | 7 | 4.158 | 0.594 | 15.526 | ***0.0001*** | 9937 |
| CaxWDxSD | 4 | 18.026 | 0.45064 | 0.79115 | 0.5379 | 9952 |  | CaxWDxSD | 4 | 0.28711 | 0.0718 | 18.761 | 0.1485 | 9949 |
| Res | 23 | 13.101 | 0.56961 |  |  |  |  | Res | 23 | 0.87994 | 0.0383 |  |  |  |
| Total | 49 | 49 |  |  |  |  |  | Total | 49 | 49 |  |  |  |  |

PERMANOVA test for canyon areas (Ca: Gollum and Whittard), water depths (WD: 700 and 1000 m), sediment depths (SD: 1, 2, 3, 4, 5 cm), cores (Co: 1-12), and interaction terms. Chl-a: chlorophyll a, CPE: chloroplastic pigment equivalents, Chl-a:phaeo: chlorophyll a divided by its degradation products (phaeopigments) indicating ‘freshness’ of the phytodetrital OM, Chl-a:TOC: chlorophyll a divided by total organic carbon content indicating bioavailability of the bulk OM; TOC: total organic carbon, TN: total nitrogen, C:N: molar carbon/nitrogen ratio. Data was normalised; resemblance was calculated using Euclidean Distance. Bold values indicate significant differences at p < 0.05, bold italic values indicate significant differences at p < 0.01.
