# Supplementary material for: Meiofauna in the Gollum Channels and the Whittard Canyon, Celtic Margin—How Local Environmental Conditions Shape Nematode Structure and Function
Source: PLoS One. 2011 May 18;6(5):e20094. doi: 10.1371/journal.pone.0020094 (PMC3097227; doi:10.1371/journal.pone.0020094)
Supplement: Table S2 — Meiofauna taxa abundances per sediment layer. Values denote means of replicates (ind. 10 cm−2). St. Dev.: standard deviation. G1000: Gollum Channel at 1090 m depth, G700: Gollum Channel at 755 m depth, W1000: Whittard Canyon at 1160 m depth, W700: Whittard Canyon at 762 m depth. (DOCX) [file pone.0020094.s003.docx]

**Table S2.** Meiofauna taxa abundances per sediment layer

| Station | G700 | | | | | | G1000 | | | | | | W700 | | | | | | W1000 | | | | | |
| --- | --- | --- | --- | --- | --- | --- | --- | --- | --- | --- | --- | --- | --- | --- | --- | --- | --- | --- | --- | --- | --- | --- | --- | --- |
| Sediment depth (cm) | 0-1 | 1-2 | 2-3 | 3-4 | 4-5 | **0-5** | 0-1 | 1-2 | 2-3 | 3-4 | 4-5 | **0-5** | 0-1 | 1-2 | 2-3 | 3-4 | 4-5 | **0-5** | 0-1 | 1-2 | 2-3 | 3-4 | 4-5 | **0-5** |
| Amphipoda | 0.3 | 0.0 | 0.0 | 0.0 | 0.0 | **0.3** | 0.5 | 0.4 | 0.0 | 0.0 | 0.0 | **0.9** | 0.0 | 0.0 | 0.0 | 0.0 | 0.0 | **0.0** | 0.0 | 0.0 | 0.0 | 0.0 | 0.0 | **0.0** |
| Aplacophora | 0.0 | 0.0 | 0.0 | 0.0 | 0.0 | **0.0** | 0.0 | 0.0 | 0.0 | 0.0 | 0.0 | **0.0** | 0.0 | 0.0 | 0.0 | 0.0 | 0.0 | **0.0** | 0.1 | 0.0 | 0.0 | 0.0 | 0.0 | **0.1** |
| Bivalvia | 1.3 | 0.0 | 0.0 | 0.0 | 0.1 | **1.4** | 1.7 | 0.7 | 0.0 | 0.0 | 0.0 | **2.4** | 0.4 | 0.5 | 0.0 | 0.0 | 0.0 | **0.9** | 0.5 | 0.1 | 0.0 | 0.0 | 0.0 | **0.7** |
| Gastropoda | 0.0 | 0.0 | 0.0 | 0.0 | 0.0 | **0.0** | 0.1 | 0.0 | 0.0 | 0.0 | 0.0 | **0.1** | 0.0 | 0.0 | 0.0 | 0.0 | 0.0 | **0.0** | 0.0 | 0.0 | 0.0 | 0.0 | 0.0 | **0.0** |
| Gastrotricha | 0.0 | 0.0 | 0.0 | 0.0 | 0.0 | **0.0** | 0.1 | 0.0 | 0.0 | 0.0 | 0.0 | **0.1** | 0.0 | 0.0 | 0.0 | 0.0 | 0.0 | **0.0** | 0.5 | 0.0 | 0.0 | 0.0 | 0.0 | **0.5** |
| Halacarida | 0.0 | 0.0 | 0.0 | 0.0 | 0.0 | **0.0** | 0.3 | 0.1 | 0.0 | 0.0 | 0.1 | **0.5** | 0.0 | 0.0 | 0.0 | 0.0 | 0.0 | **0.0** | 0.0 | 0.0 | 0.0 | 0.0 | 0.0 | **0.0** |
| Copepoda | 16.6 | 2.2 | 0.7 | 0.1 | 0.1 | **19.7** | 13.2 | 1.4 | 0.0 | 0.4 | 0.1 | **15.2** | 24.8 | 4.8 | 0.8 | 0.1 | 0.4 | **31.0** | 9.3 | 1.4 | 1.8 | 0.8 | 0.0 | **13.3** |
| Holothuroidea | 0.0 | 0.0 | 0.0 | 0.0 | 0.0 | **0.0** | 0.0 | 0.1 | 0.0 | 0.0 | 0.0 | **0.1** | 0.0 | 0.0 | 0.0 | 0.0 | 0.0 | **0.0** | 0.0 | 0.0 | 0.0 | 0.0 | 0.0 | **0.0** |
| Isopoda | 0.1 | 0.0 | 0.0 | 0.0 | 0.0 | **0.1** | 0.0 | 0.0 | 0.0 | 0.0 | 0.0 | **0.0** | 0.0 | 0.0 | 0.0 | 0.0 | 0.0 | **0.0** | 0.0 | 0.0 | 0.0 | 0.0 | 0.0 | **0.0** |
| Kinorrhyncha | 1.6 | 0.0 | 0.1 | 0.0 | 0.0 | **1.7** | 1.3 | 0.1 | 0.0 | 0.0 | 0.0 | **1.4** | 2.0 | 0.4 | 0.0 | 0.0 | 0.0 | **2.4** | 0.0 | 0.0 | 0.0 | 0.0 | 0.0 | **0.0** |
| Nauplii | 27.3 | 2.1 | 0.3 | 0.7 | 0.0 | **30.3** | 20.4 | 2.5 | 1.4 | 0.5 | 0.5 | **25.3** | 40.9 | 4.2 | 0.0 | 0.0 | 0.3 | **45.3** | 13.2 | 2.1 | 0.8 | 0.0 | 0.0 | **16.1** |
| Nematoda | 463.5 | 407.8 | 152.1 | 28.9 | 28.2 | **1080.4** | 514.3 | 510.2 | 206.5 | 75.4 | 48.3 | **1354.8** | 382.5 | 275.1 | 142.1 | 86.5 | 64.1 | **950.3** | 560.8 | 280.2 | 97.6 | 41.3 | 34.9 | **1014.7** |
| Oligochaeta | 0.7 | 0.1 | 0.0 | 0.1 | 0.0 | **0.9** | 0.3 | 0.8 | 0.3 | 0.0 | 0.0 | **1.3** | 0.1 | 0.1 | 0.5 | 0.3 | 0.1 | **1.2** | 0.3 | 0.0 | 0.0 | 0.0 | 0.0 | **0.3** |
| Ostracoda | 1.0 | 0.0 | 0.1 | 0.0 | 0.0 | **1.2** | 1.8 | 0.1 | 0.1 | 0.3 | 0.0 | **2.4** | 1.2 | 0.3 | 0.0 | 0.0 | 0.0 | **1.4** | 0.5 | 0.1 | 0.0 | 0.0 | 0.0 | **0.7** |
| Polychaeta | 1.7 | 2.2 | 0.7 | 0.7 | 0.8 | **6.0** | 2.9 | 2.4 | 0.4 | 0.5 | 0.3 | **6.4** | 2.9 | 2.4 | 2.2 | 0.5 | 0.7 | **8.6** | 1.8 | 1.3 | 0.5 | 0.1 | 0.4 | **4.2** |
| Rotifera | 2.2 | 3.1 | 2.4 | 0.9 | 0.5 | **9.1** | 3.7 | 1.6 | 0.8 | 1.6 | 0.5 | **8.1** | 2.9 | 1.8 | 1.8 | 0.8 | 1.4 | **8.8** | 2.9 | 2.0 | 2.0 | 1.2 | 2.9 | **10.8** |
| Tanaidiacea | 0.5 | 0.0 | 0.0 | 0.0 | 0.0 | **0.5** | 0.3 | 0.0 | 0.0 | 0.0 | 0.0 | **0.3** | 0.0 | 0.1 | 0.0 | 0.1 | 0.0 | **0.3** | 0.3 | 0.1 | 0.0 | 0.0 | 0.0 | **0.4** |
| Tantulocarida | 0.1 | 0.0 | 0.0 | 0.0 | 0.0 | **0.1** | 0.0 | 0.0 | 0.0 | 0.0 | 0.0 | **0.0** | 0.5 | 0.0 | 0.0 | 0.0 | 0.0 | **0.5** | 0.1 | 0.0 | 0.0 | 0.0 | 0.0 | **0.1** |
| Tardigrada | 5.5 | 0.1 | 0.0 | 0.0 | 0.1 | **5.7** | 5.2 | 0.3 | 0.1 | 0.3 | 0.0 | **5.9** | 2.6 | 0.4 | 0.0 | 0.0 | 0.0 | **3.0** | 3.3 | 0.9 | 0.0 | 0.0 | 0.0 | **4.2** |
| Turbellaria | 0.0 | 0.0 | 0.0 | 0.0 | 0.0 | **0.0** | 0.0 | 0.1 | 0.0 | 0.1 | 0.0 | **0.3** | 0.1 | 0.0 | 0.0 | 0.0 | 0.0 | **0.1** | 0.1 | 0.0 | 0.0 | 0.0 | 0.0 | **0.1** |
| Echinodermata | 0.0 | 0.0 | 0.0 | 0.0 | 0.0 | **0.0** | 0.1 | 0.0 | 0.0 | 0.0 | 0.0 | **0.1** | 0.0 | 0.0 | 0.0 | 0.0 | 0.0 | **0.0** | 0.0 | 0.0 | 0.0 | 0.0 | 0.0 | **0.0** |
| Total | 522.4 | 417.8 | 156.2 | 31.4 | 29.9 | 1157.6 | 566.1 | 520.8 | 209.7 | 79.0 | 49.9 | 1425.6 | 460.9 | 290.1 | 147.5 | 88.3 | 67.0 | 1053.8 | 593.7 | 288.3 | 102.7 | 43.4 | 38.1 | 1066.2 |
| St. Dev. | 50.2 | 82.3 | 35.2 | 28.2 | 1.6 | 109.5 | 47.6 | 106.3 | 155.1 | 20.2 | 17.5 | 267.2 | 283.7 | 148.8 | 77.2 | 26.2 | 15.4 | 419.7 | 135.3 | 71.6 | 47.4 | 25.1 | 15.9 | 277.8 |

Values denote means of replicates (ind. 10 cm^-2^). St. Dev.: standard deviation. G1000: Gollum Channel at 1090 m depth, G700: Gollum Channel at 755 m depth, W1000: Whittard Canyon at 1160 m depth, W700: Whittard Canyon at 762 m depth
