# Supplementary material for: Meiofauna in the Gollum Channels and the Whittard Canyon, Celtic Margin—How Local Environmental Conditions Shape Nematode Structure and Function
Source: PLoS One. 2011 May 18;6(5):e20094. doi: 10.1371/journal.pone.0020094 (PMC3097227; doi:10.1371/journal.pone.0020094)
Supplement: Table S3 — Results from pair-wise multivariate PERMANOVA analyses for differences in nematode community structure. PERMANOVA pair-wise comparisons within each of the Ca×WD×SD levels as part of the repeated measures analysis, including Monte-Carlo permutation p values (P(MC)). The pair-wise comparisons give an indication of the individual contribution to the significant three-way interaction term (Ca×WD×SD) with differences between canyon areas (Ca: Gollum and Whittard), water depths (WD: 700 and 1000 m), sediment depths (SD: 1, 2, 3, 4, 5 cm). Data was standardised and square root transformed; resemblance was calculated using Bray-Curtis. Bold values indicate significant differences at p <0.05. (DOCX) [file pone.0020094.s004.docx]

**Table S3.** Results from pair-wise multivariate PERMANOVA analyses for differences in nematode community structure

| Within Ca x SD | WD comparisons | t | P(perm) | perms | P(MC) |
| --- | --- | --- | --- | --- | --- |
| G x 0-1 | 1000, 700 | 10.982 | 0.274 | 10 | 0.359 |
| G x 1-2 | 1000, 700 | 11.462 | 0.202 | 10 | 0.292 |
| G x 2-3 | 1000, 700 | 13.299 | 0.117 | 10 | 0.161 |
| G x 3-4 | 1000, 700 | 1.29 | 0.207 | 10 | 0.254 |
| G x 4-5 | 1000, 700 | 12.648 | 0.236 | 10 | 0.223 |
| W x 0-1 | 1000, 700 | 17.722 | 0.101 | 10 | **0.036** |
| W x 1-2 | 1000, 700 | 16.623 | 0.102 | 10 | 0.07 |
| W x 2-3 | 1000, 700 | 21.838 | 0.098 | 10 | **0.03** |
| W x 3-4 | 1000, 700 | 23.051 | 0.117 | 10 | **0.021** |
| W x 4-5 | 1000, 700 | 2.195 | 0.111 | 10 | **0.026** |
| Within WD x SD | Ca comparisons | t | P(perm) | perms | P(MC) |
| 1000 x 0-1 | G, W | 17.076 | 0.1 | 10 | 0.065 |
| 1000 x 1-2 | G, W | 19.605 | 0.11 | 10 | **0.038** |
| 1000 x 2-3 | G, W | 20.428 | 0.099 | 10 | **0.048** |
| 1000 x 3-4 | G, W | 17.742 | 0.105 | 10 | 0.062 |
| 1000 x 4-5 | G, W | 14.656 | 0.106 | 10 | 0.119 |
| 1000 x 0-1 | G, W | 13.361 | 0.104 | 10 | 0.168 |
| 1000 x 1-2 | G, W | 15.105 | 0.096 | 10 | 0.099 |
| 1000 x 2-3 | G, W | 15.766 | 0.079 | 10 | 0.101 |
| 1000 x 3-4 | G, W | 18.387 | 0.1 | 10 | 0.074 |
| 1000 x 4-5 | G, W | 19.686 | 0.111 | 10 | **0.033** |
| Within Ca x WD | SD comparisons | t | P(perm) | Unique perms | P(MC) |
| G x 1000 | 0-1, 1-2 | 19.134 | 0.091 | 38 | 0.106 |
| G x 1000 | 0-1, 2-3 | 16.692 | 0.149 | 38 | 0.166 |
| G x 1000 | 0-1, 3-4 | 19.479 | 0.12 | 38 | 0.112 |
| G x 1000 | 0-1, 4-5 | 18.205 | 0.15 | 38 | 0.125 |
| G x 1000 | 1-2, 2-3 | 12.109 | 0.292 | 38 | 0.342 |
| G x 1000 | 1-2, 3-4 | 13.972 | 0.243 | 38 | 0.247 |
| G x 1000 | 1-2, 4-5 | 18.519 | 0.131 | 38 | 0.125 |
| G x 1000 | 2-3, 3-4 | 11.605 | 0.319 | 38 | 0.35 |
| G x 1000 | 2-3, 4-5 | 15.228 | 0.2 | 38 | 0.188 |
| G x 1000 | 3-4, 4-5 | 1.169 | 0.317 | 38 | 0.336 |
| G x 700 | 0-1, 1-2 | 15.988 | 0.205 | 38 | 0.166 |
| G x 700 | 0-1, 2-3 | 16.121 | 0.181 | 38 | 0.174 |
| G x 700 | 0-1, 3-4 | 15.077 | 0.3436 | 15 | 0.321 |
| G x 700 | 0-1, 4-5 | 17.176 | 0.13 | 38 | 0.164 |
| G x 700 | 1-2, 2-3 | 13.086 | 0.254 | 38 | 0.257 |
| G x 700 | 1-2, 3-4 | 15.316 | 0.3251 | 15 | 0.293 |
| G x 700 | 1-2, 4-5 | 15.194 | 0.223 | 38 | 0.196 |
| G x 700 | 2-3, 3-4 | 0.97901 | 0.6139 | 15 | 0.515 |
| G x 700 | 2-3, 4-5 | 11.434 | 0.327 | 38 | 0.362 |
| G x 700 | 3-4, 4-5 | 0.98234 | 0.6699 | 15 | 0.51 |
| W x 1000 | 0-1, 1-2 | 11.137 | 0.361 | 38 | 0.373 |
| W x 1000 | 0-1, 2-3 | 21.239 | 0.093 | 38 | 0.085 |
| W x 1000 | 0-1, 3-4 | 19.017 | 0.103 | 38 | 0.112 |
| W x 1000 | 0-1, 4-5 | 19.199 | 0.11 | 38 | 0.117 |
| W x 1000 | 1-2, 2-3 | 15.611 | 0.179 | 38 | 0.176 |
| W x 1000 | 1-2, 3-4 | 15.535 | 0.218 | 38 | 0.165 |
| W x 1000 | 1-2, 4-5 | 17.449 | 0.127 | 38 | 0.141 |
| W x 1000 | 2-3, 3-4 | 10.537 | 0.336 | 38 | 0.434 |
| W x 1000 | 2-3, 4-5 | 13.275 | 0.266 | 38 | 0.272 |
| W x 1000 | 3-4, 4-5 | 13.309 | 0.238 | 38 | 0.259 |
| W x 700 | 0-1, 1-2 | 15.777 | 0.169 | 38 | 0.187 |
| W x 700 | 0-1, 2-3 | 20.172 | 0.095 | 38 | 0.105 |
| W x 700 | 0-1, 3-4 | 31.011 | 0.114 | 38 | 0.043 |
| W x 700 | 0-1, 4-5 | 27.385 | 0.094 | 38 | 0.052 |
| W x 700 | 1-2, 2-3 | 13.854 | 0.237 | 38 | 0.259 |
| W x 700 | 1-2, 3-4 | 17.235 | 0.114 | 38 | 0.126 |
| W x 700 | 1-2, 4-5 | 20.178 | 0.098 | 38 | 0.109 |
| W x 700 | 2-3, 3-4 | 12.004 | 0.311 | 38 | 0.295 |
| W x 700 | 2-3, 4-5 | 13.678 | 0.23 | 38 | 0.265 |
| W x 700 | 3-4, 4-5 | 11.521 | 0.286 | 38 | 0.337 |

PERMANOVA pair-wise comparisons within each of the Ca x WD x SD levels as part of the repeated measures analysis, including Monte-Carlo permutation p values (P(MC)). The pair-wise comparisons give an indication of the individual contribution to the significant three-way interaction term (Ca x WD x SD) with differences between canyon areas (Ca: Gollum and Whittard), water depths (WD: 700 and 1000 m), sediment depths (SD: 1, 2, 3, 4, 5 cm). Data was standardised and square root transformed; resemblance was calculated using Bray-Curtis. Bold values indicate significant differences at p < 0.05.
