# Supplementary material for: Meiofauna in the Gollum Channels and the Whittard Canyon, Celtic Margin—How Local Environmental Conditions Shape Nematode Structure and Function
Source: PLoS One. 2011 May 18;6(5):e20094. doi: 10.1371/journal.pone.0020094 (PMC3097227; doi:10.1371/journal.pone.0020094)
Supplement: Table S4 — Spearman correlation coefficients between environmental variables. Chl-a: chlorophyll a, CPE: chloroplastic pigment equivalents, Chl-a∶phaeo: chlorophyll a divided by its degradation products (phaeophytines) indicating ‘freshness’ of the phytodetrital OM, TN: total nitrogen content, TOC: total organic carbon content, C∶N: molar carbon-nitrogen ratio, Chl-a∶TOC: chlorophyll a divided by total organic carbon content indicating bioavailability of the bulk OM, CPE∶TOC: total pigment derived matter relative to the bulk OM. (DOCX) [file pone.0020094.s005.docx]

| Spearman corr. (-1 to 1) | log(chl-a+0.1) | CPE | log(chl-a:phaeo+0.1) | TN | TOC | C:N | log(chl-a:TOC+0.1) | log(CPE:TOC+0.1) |
| --- | --- | --- | --- | --- | --- | --- | --- | --- |
| CPE | 0.834 |  |  |  |  |  |  |  |
| log(chl-a:phaeo+0.1) | 0.755 | 0.760 |  |  |  |  |  |  |
| N% | 0.641 | 0.899 | 0.581 |  |  |  |  |  |
| C% | 0.304 | 0.624 | 0.290 | 0.740 |  |  |  |  |
| C:N | -0.109 | 0.067 | -0.079 | 0.114 | 0.705 |  |  |  |
| log(chl-a:TOC+0.1) | 0.876 | 0.682 | 0.805 | 0.419 | 0.086 | -0.218 |  |  |
| log(CPE:TOC+0.1) | 0.693 | 0.851 | 0.821 | 0.683 | 0.383 | -0.091 | 0.778 |  |
| Mean grain size | -0.497 | -0.723 | -0.402 | -0.850 | -0.739 | -0.232 | -0.308 | -0.558 |

**Table S4**. Spearman correlation coefficients between environmental variables

Chl-a: chlorophyll a, CPE: chloroplastic pigment equivalents, Chl-a:phaeo: chlorophyll a divided by its degradation products (phaeophytines) indicating ‘freshness’ of the phytodetrital OM, TN: total nitrogen content, TOC: total organic carbon content, C:N: molar carbon-nitrogen ratio, Chl-a:TOC: chlorophyll a divided by total organic carbon content indicating bioavailability of the bulk OM, CPE:TOC: total pigment derived matter relative to the bulk OM, Mean grain size: volume weighted mean grain size.
