# Supplementary material for: Meiofauna in the Gollum Channels and the Whittard Canyon, Celtic Margin—How Local Environmental Conditions Shape Nematode Structure and Function
Source: PLoS One. 2011 May 18;6(5):e20094. doi: 10.1371/journal.pone.0020094 (PMC3097227; doi:10.1371/journal.pone.0020094)
Supplement: Table S5 — Distance-based linear model (DISTLM) for genera assemblages and selected environmental variables. Variables: Selected environmental variables used to calculate the optimum model. Marginal tests: explanation of variation for each variable taken alone. Sequential tests: conditional tests of individual variables in constructing the model. Each test examines whether adding the variable contributes significantly to the explained variation. Selection procedure: step-wise, selection criterion: adjusted R2. Prop.: % variation explained. Cumul.: cumulative variation explained. Chl-a: chlorophyll a, CPE: chloroplastic pigment equivalents, Chl-a∶phaeo: chlorophyll a divided by its degradation products (phaeophytines) indicating ‘freshness’ of the phytodetrital OM, TN: total nitrogen content, TOC: total organic carbon content, C∶N: molar carbon-nitrogen ratio, Chl-a∶TOC: chlorophyll a divided by total organic carbon content indicating bioavailability of the bulk OM, CPE∶TOC: total pigment derived matter relative to the bulk OM, Mean grain size: volume weighted mean grain size. (DOCX) [file pone.0020094.s006.docx]

| VARIABLES | |  | MARGINAL TESTS | | | | |
| --- | --- | --- | --- | --- | --- | --- | --- |
| 1 | log(chl-a+0.1) |  | Variable | SS(trace) | Pseudo-F | P | Prop. |
| 2 | CPE |  | log(chl-a+0.1) | 62.85 | 18.891 | 0.0383 | 0.0950 |
| 3 | log(chl-a:phaeo+0.1) |  | CPE | 78.796 | 24.332 | 0.0155 | 0.1191 |
| 4 | TN |  | log(chl-a:phaeo+0.1) | 73.881 | 22.623 | 0.0248 | 0.1117 |
| 5 | TOC |  | TN | 93.666 | 29.681 | 0.0054 | 0.1416 |
| 6 | C:N |  | TOC | 85.248 | 26.619 | 0.0113 | 0.1288 |
| 7 | log(chl-a:TOC+0.1) |  | C/N | 39.263 | 11.354 | 0.3022 | 0.0593 |
| 8 | log(CPE:TOC+0.1) |  | log(chl-a:TOC+0.1) | 85.444 | 26.689 | 0.0095 | 0.1291 |
| 9 | Mean grain size |  | log(CPE:TOC+0.1) | 83.178 | 2.588 | 0.0131 | 0.1257 |
|  |  |  | Mean grain size | 80.058 | 24.775 | 0.0152 | 0.1210 |
|  |  |  |  |  |  |  |  |
| SEQUENTIAL TESTS (Best model construction) | | | | | | | |
| Variable | Adjusted R² | SS(trace) | Pseudo-F | P | Prop. | Cumul. | res.df |
| +TN | 0.0938 | 93.666 | 29.681 | 0.0042 | 0.1416 | 0.14155 | 18 |
| +log(chl-a:TOC+0.1) | 0.2297 | 111.99 | 41.746 | 0.0001 | 0.1693 | 0.3108 | 17 |
| +Mean grain size | 0.3158 | 74.8 | 31.392 | 0.0005 | 0.1130 | 0.42384 | 16 |
| +Log(chla+0.1) | 0.3511 | 42.257 | 18.699 | 0.0353 | 0.0639 | 0.4877 | 15 |
| +Log(chl-a:phaeo+0.1) | 0.3619 | 27.856 | 12.534 | 0.2272 | 0.0421 | 0.5298 | 14 |
| +CPE | 0.3631 | 22.776 | 10.268 | 0.407 | 0.0344 | 0.56422 | 13 |
| +C/N | 0.3704 | 25.241 | 11.512 | 0.3147 | 0.0381 | 0.60236 | 12 |
| +TOC | 0.3840 | 27.117 | 12.639 | 0.2463 | 0.0410 | 0.64334 | 11 |

**Table S5.** Distance-based linear model (DISTLM) for genera assemblages and selected environmental variables

Variables: Selected environmental variables used to calculate the optimum model. Marginal tests: explanation of variation for each variable taken alone. Sequential tests: conditional tests of individual variables in constructing the model. Each test examines whether adding the variable contributes significantly to the explained variation. Selection procedure: step-wise, selection criterion: adjusted R². Prop.: % variation explained. Cumul.: cumulative variation explained. Chl-a: chlorophyll a, CPE: chloroplastic pigment equivalents, Chl-a:phaeo: chlorophyll a divided by its degradation products (phaeophytines) indicating ‘freshness’ of the phytodetrital OM, TN: total nitrogen content, TOC: total organic carbon content, C:N: molar carbon-nitrogen ratio, Chl-a:TOC: chlorophyll a divided by total organic carbon content indicating bioavailability of the bulk OM, CPE:TOC: total pigment derived matter relative to the bulk OM, Mean grain size: volume weighted mean grain size.
