# Supplementary material for: Meiofauna in the Gollum Channels and the Whittard Canyon, Celtic Margin—How Local Environmental Conditions Shape Nematode Structure and Function
Source: PLoS One. 2011 May 18;6(5):e20094. doi: 10.1371/journal.pone.0020094 (PMC3097227; doi:10.1371/journal.pone.0020094)
Supplement: Table S6 — Distance-based linear models (DISTLM) - Sequential tests for best fitting model for each of the univariate biotic parameters. Sequential tests: conditional tests of individual variables in constructing the model. Each test examines whether adding the variable contributes significantly to the explained variation. Selection procedure: step-wise, selection criterion: adjusted R2. Prop.: % variation explained. Cumul.: cumulative variation explained. NemaDens: nematode abundance, Chemos. RA: relative abundance of the chemosynthetic Astomonema nematodes, NemaBiom: total nematode biomass, Chemos. Biomass: total biomass of the chemosynthetic Astomonema nematodes, TD: trophic diversity, H0: genus richness, H1: Hill's diversity index, EG(51): expected number of genera. Chl-a: chlorophyll a, CPE: chloroplastic pigment equivalents, Chl-a∶phaeo: chlorophyll a divided by its degradation products (phaeophytines) indicating ‘freshness’ of the phytodetrital OM, TN: total nitrogen content, TOC: total organic carbon content, C∶N: molar carbon-nitrogen ratio, Chl-a∶TOC: chlorophyll a divided by total organic carbon content indicating bioavailability of the bulk OM, CPE∶TOC: total pigment derived matter relative to the bulk OM, Mean grain size: volume weighted mean grain size. (DOCX) [file pone.0020094.s007.docx]

| **NemaDens** | | | | | | | |
| --- | --- | --- | --- | --- | --- | --- | --- |
| Variable | Adj R² | SS(trace) | Pseudo-F | P | Prop. | Cumul. | res.df |
| +log(chl-a:phaeo+0.1) | 0.31435 | 239170 | 97109 | 0.0077 | 0.3504 | 0.3504 | 18 |
| +Mean grain size | 0.47635 | 123550 | 65688 | 0.0214 | 0.1810 | 0.5315 | 17 |
| +log(CPE:TOC+0.1) | 0.56502 | 69769 | 44654 | 0.0523 | 0.1022 | 0.6337 | 16 |
| **Chemos. RA** |  |  |  |  |  |  |  |
| Variable | Adj R² | SS(trace) | Pseudo-F | P | Prop. | Cumul. | res.df |
| +TOC | 0.3375 | 0.1123 | 10679 | 0.0065 | 0.3724 | 0.3724 | 18 |
| +log(chla+0.1) | 0.42383 | 0.0338 | 36968 | 0.0887 | 0.1121 | 0.4845 | 17 |
| +TN | 0.57645 | 0.0479 | 71256 | 0.0193 | 0.1589 | 0.6433 | 16 |
| +log(chl-a:TOC+0.1) | 0.57669 | 0.0068 | 10094 | 0.3129 | 0.0225 | 0.6658 | 15 |
| +log(chl-a:phaeo+0.1) | 0.61474 | 0.0152 | 24813 | 0.1368 | 0.0530 | 0.7161 | 14 |
| **Biomass** | | | | | | | |
| Variable | Adj R² | SS(trace) | Pseudo-F | P | Prop. | Cumul. | res.df |
| +log(chl-a:phaeo+0.1) | 0.03819 | 2186.2 | 17543 | 0.1969 | 0.0888 | 0.0888 | 18 |
| +Mean grain size | 0.19763 | 4758 | 4577 | 0.0485 | 0.1933 | 0.2821 | 17 |
| +log(chl-a:TOC+0.1) | 0.23187 | 1749.2 | 17577 | 0.2075 | 0.0711 | 0.3532 | 16 |
| +log(CPE:TOC+0.1) | 0.27116 | 1758.9 | 18626 | 0.1936 | 0.0715 | 0.4246 | 15 |
| **Chemos. Biomass** | | | | | | | |
| Variable | Adj R² | SS(trace) | Pseudo-F | P | Prop. | Cumul. | res.df |
| +TOC | 0.4238 | 10.442 | 14974 | 0.0027 | 0.4541 | 0.4541 | 18 |
| +log(chla+0.1) | 0.45822 | 14.056 | 21437 | 0.1672 | 0.0611 | 0.5153 | 17 |
| +TN | 0.68142 | 49.776 | 13 | 0.0032 | 0.2165 | 0.7317 | 16 |
| +log(CPE:TOC+0.1) | 0.69125 | 0.56413 | 15098 | 0.2410 | 0.0245 | 0.7563 | 15 |
| **TD** | | | | | | | |
| Variable | Adj R² | SS(trace) | Pseudo-F | P | Prop. | Cumul. | res.df |
| +TN | 0.12809 | 0.68233 | 37913 | 0.0683 | 0.1740 | 0.1740 | 18 |
| ***H_0_*** | | | | | | | |
| Variable | Adj R² | SS(trace) | Pseudo-F | P | Prop. | Cumul. | res.df |
| +log(chl-a:phaeo+0.1) | 0.22144 | 1107.6 | 64041 | 0.0221 | 0.2624 | 0.2624 | 18 |
| +Mean grain size | 0.35639 | 682.54 | 47741 | 0.0427 | 0.1617 | 0.4241 | 17 |
| +TN | 0.55924 | 863.93 | 8824 | 0.0096 | 0.2047 | 0.6288 | 16 |
| ***H_1_*** | | | | | | | |
| Variable | Adj R² | SS(trace) | Pseudo-F | P | Prop. | Cumul. | res.df |
| +log(chl-a:phaeo+0.1) | 0.20446 | 703.88 | 58832 | 0.0271 | 0.2463 | 0.2463 | 18 |
| +Mean grain size | 0.31816 | 410.33 | 40015 | 0.0590 | 0.1436 | 0.3899 | 17 |
| +TN | 0.4644 | 454.45 | 56418 | 0.0289 | 0.1590 | 0.5490 | 16 |
| **EG(51)** | | | | | | | |
| Variable | Adj R² | SS(trace) | Pseudo-F | P | Prop. | Cumul. | res.df |
| +log(chla+0.1) | 0.16041 | 178.96 | 5 | 0.0627 | 0.2046 | 0.2046 | 18 |
| +TOC | 0.31969 | 163.31 | 52142 | 0.0381 | 0.1867 | 0.3913 | 17 |
| +log(CPE:TOC+0.1) | 0.32419 | 34.635 | 11132 | 0.2984 | 0.0396 | 0.4309 | 16 |

**Table S6.** Distance-based linear models (DISTLM) - Sequential tests for best fitting model for each of the univariate biotic parameters.

Sequential tests: conditional tests of individual variables in constructing the model. Each test examines whether adding the variable contributes significantly to the explained variation. Selection procedure: step-wise, selection criterion: adjusted R². Prop.: % variation explained. Cumul.: cumulative variation explained. NemaDens: nematode abundance, Chemos. RA: relative abundance of the chemosynthetic *Astomonema* nematodes, NemaBiom: total nematode biomass, Chemos. Biomass: total biomass of the chemosynthetic *Astomonema* nematodes, TD: trophic diversity, *H_0_*: genus richness, *H_1_*: Hill’s diversity index, EG(51): expected number of genera. Chl-a: chlorophyll a, CPE: chloroplastic pigment equivalents, Chl-a:phaeo: chlorophyll a divided by its degradation products (phaeophytines) indicating ‘freshness’ of the phytodetrital OM, TN: total nitrogen content, TOC: total organic carbon content, C:N: molar carbon-nitrogen ratio, Chl-a:TOC: chlorophyll a divided by total organic carbon content indicating bioavailability of the bulk OM, CPE:TOC: total pigment derived matter relative to the bulk OM, Mean grain size: volume weighted mean grain size.
